# Arrest of Nuclear Division in *Plasmodium* through Blockage of Erythrocyte Surface Exposed Ribosomal Protein P2

**DOI:** 10.1371/journal.ppat.1002858

**Published:** 2012-08-09

**Authors:** Sudipta Das, Himanish Basu, Reshma Korde, Rita Tewari, Shobhona Sharma

**Affiliations:** 1 Department of Biological Sciences, Tata Institute of Fundamental Research, Mumbai, India; 2 Centre for Genetics and Genomics, Queen's Medical Centre, The University of Nottingham, Nottingham, United Kingdom; Seattle Biomedical Research Institute, United States of America

## Abstract

Malaria parasites reside inside erythrocytes and the disease manifestations are linked to the growth inside infected erythrocytes (IE). The growth of the parasite is mostly confined to the trophozoite stage during which nuclear division occurs followed by the formation of cell bodies (schizogony). The mechanism and regulation of schizogony are poorly understood. Here we show a novel role for a *Plasmodium falciparum* 60S stalk ribosomal acidic protein P2 (PfP2) (PFC0400w), which gets exported to the IE surface for 6–8 hrs during early schizogony, starting around 26–28 hrs post-merozoite invasion. The surface exposure is demonstrated using multiple PfP2-specific monoclonal antibodies, and is confirmed through transfection using PfP2-GFP. The IE surface-exposed PfP2-protein occurs mainly as SDS-resistant P2-homo-tetramers. Treatment with anti-PfP2 monoclonals causes arrest of IEs at the first nuclear division. Upon removal of the antibodies, about 80–85% of synchronized parasites can be released even after 24 hrs of antibody treatment. It has been reported that a tubovesicular network (TVN) is set up in early trophozoites which is used for nutrient import. Anti-P2 monoclonal antibodies cause a complete fragmentation of TVN by 36 hrs, and impairs lipid import in IEs. These may be downstream causes for the cell-cycle arrest. Upon antibody removal, the TVN is reconstituted, and the cell division progresses. Each of the above properties is observed in the rodent malaria parasite species *P. yoelii* and *P. berghei*. The translocation of the P2 protein to the IE surface is therefore likely to be of fundamental importance in *Plasmodium* cell division.

## Author Summary

Malaria is a serious human health problem and the disease manifestations are caused by erythrocytic stages. The growth of *Plasmodium* in erythrocytes occurs through the peculiar process of schizogony where the nucleus undergoes several divisions followed by cell body formation. Inside a red blood cell (RBC), how does the parasite know when to start the cell division? Is there an influence from the external milieu? Here we report a novel involvement of a *Plasmodium* ribosomal protein P2 in this process. P2 gets trafficked to the infected erythrocyte surface at the onset of cell division for 6–8 hrs. Detergent resistant P2-tetramer is observed on erythrocyte surface and such an oligomerization occurs in the parasite just prior to the cell division. Blocking P2 on erythrocyte surface using monoclonal antibodies causes an arrest of parasites at the first nuclear division. Removal of antibodies reverses the arrest and parasites progress through cell division. Earlier reports have documented that a tubovesicular network (TVN), required for nutrient uptake, develops in early trophozoite stage of the parasites. Treatment with anti-P2-antibodies causes disintegration of TVN, resulting in impaired lipid import, which may be the eventual cause of the cell-cycle arrest. A study of this process of P2 oligomerization and elucidating the role of P2 on the infected erythrocyte surface at a specific parasite developmental stage might uncover novel regulations operating at the start of cell division in *Plasmodium*.

## Introduction

Malaria, caused by the species *Plasmodium*, is a prevalent tropical infectious disease, which continues to take a large toll on lives (WHO guidelines, 2008). The malarial pathology is largely caused by the cyclic growth of the parasite within the infected erythrocytes (IE). Very little is known about molecular mechanisms of replication of *Plasmodium* in the erythrocytes. Cell division in *Plasmodium* occurs largely through a schizogonic process, in which the nuclei divide asynchronously at first into about 16–24 nuclei, followed by the formation of cell bodies [1]–[5]. Unlike classical eukaryotic cell division, the nuclear cell membrane appears to remain intact during such divisions. Similar nuclear division without cytokinesis occurs in *Drosophila* embryonic syncytial divisions [6], [7]. It is possible that such a form of cell division is favoured when rapid eukaryotic cell division is required, such as in the case of *Drosophila* embryo. Certain filament forming fungal species also form multinuclear hyphal compartments, in which the nuclear position and cell cycle is only loosely coordinated with septum placement [8], [9]. This type of nuclear division might enable cells to spatially restrict mitoses within a shared cytoplasm, potentially facilitating local responses to nutrients or other environmental stimuli.

The entry of a cell into division in eukaryotic animal cells is controlled at two steps, the G0 to G1 transition, as also within the G1stage through a restriction (R) or ‘Start’ stage [10], [11]. The G0 to G1 signals are relevant for those cells that have been quiescent and need to metabolically become active and enter cell division. The R point, first proposed by Pardee, occurs in mid-late G1 phase [12]. This is the phase when the growth depends on the exposure to specific signals received over an extended period of time. If the signals, largely of extracellular origin, are cumulatively favourable for growth, then the cell will decide to proceed and will pass through the R point. Alternatively, the cell may halt its advance through G1 phase. Eventually the cell may exit the cell cycle, proceeding either back into G0 phase or into a post-mitotic, possibly more differentiated state. The eukaryotic cells also undergo internal checkpoint controls [13]–[16]. During a checkpoint, the cell ascertains that its metabolic household is in order, that its genome is intact, and that previous steps in its cell cycle have been executed properly before it moves ahead. Molecular players in both these steps have been worked out to great details in animal cells [17]–[20].

Virtually nothing is known about the start of the nuclear division in *Plasmodium*, or check-point controls in schizogony. Certain cell division components such as cyclin-dependent protein kinases (CDKs) and cyclins have been reported in *Plasmodium falciparum* and it is apparent that there are important divergences in the composition and properties of these components of cell cycle machinery in *Plasmodium* [5], [21]–[23]. Markers such as centrin protein PfCEN3 and Aurora-A-related kinase, PfArk1, are reported to localize to mitotic spindle poles during schizogony, but their roles are not yet well understood [23], [24]. Recent studies in the replication of *Plasmodium* have been controversial as to whether the cells go through the classical G1, S, G2, M phases; however, the asynchrony in nuclear divisions appears to favor the G1 phase followed by alternation between independent S and M phases for each nucleus [4], [5], [25].

In an earlier differential immunoscreen we have identified *Plasmodium falciparum* ribosomal protein PfP0 as a protective protein during the asexual erythrocytic stages [26], [27]. The P0 protein appears to play a pleiotropic role, since it gets surface localized to the merozoites, and seems to play a role during invasion of red cells [27]–[30]. The ribosomal role of PfP0 has also been confirmed through complementation studies in a conditional knock-out of yeast ScP0 strain using PfP0 [31]. In eukaryotic ribosomes, P0 occurs as a complex with two other small acidic ribosomal proteins (P1 and P2) [32]–[34]. *Plasmodium* contains one gene each for P1 and P2 proteins, which possess a homologous carboxy-terminal domain as is observed in yeast and human P-proteins [PlasmoDB, 35], Figure S1. A pentameric complex [(P1–P2) P0 (P1–P2)] constitutes the stalk of the large ribosomal subunit, which seems to play a role in the GTPase elongation centre [36], [37]. P1/P2 are the only ribosomal proteins that have been documented to exist in a cytoplasmic pool, and *Neisseria gonorrhoeae* P2 orthologue has been shown to exist at the cell surface and play a role in invasion [38].

In an attempt to assess the properties of the three P-proteins of *Plasmodium*, the P-proteins were expressed in *E. coli* and specific reagents were generated. In this paper we report a novel involvement of the *Plasmodium* ribosomal P2 protein at the onset of cell division. We document a translocation of P2 protein, but not P0 or P1 proteins, to the infected erythrocyte surface during early cell division. We also observe an unusual cell-cycle arrest of *Plasmodium* at the first nuclear division, when treated with a panel of anti-P2-specific monoclonal antibodies (anti-P2-mAbs). Upon removal of anti-P2-mAbs, such an arrest was released in about 80% of cells even after 24 hrs of antibody treatment. The anti-P2-mAb treatment causes a disintegration of the tubovesicular network in the parasite-infected cells and results in an impaired lipid import, which may be one of the downstream causes for the cell-cycle arrest.

## Results

### Surface localization of *Plasmodium* P2 protein on infected erythrocytes (IE)

**Figure 1:**
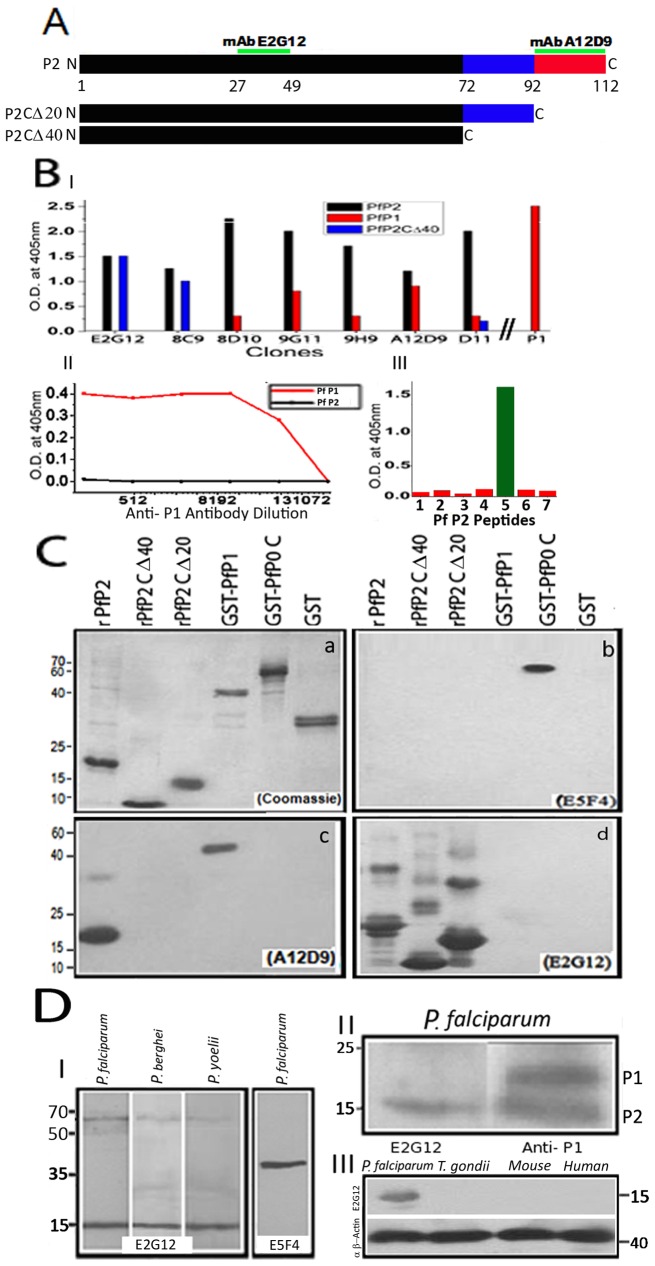
Reactivities of antibodies to P-proteins. (**A**) Schematic of the full length *P. falciparum* P2 protein and different deletion constructs (PfP2CΔ20, PfP2CΔ40). The epitope positions of P2, where the monoclonals E2G12 and A12D9 map, are shown. (**B**) **I:** ELISA using 100 ng of each recombinant PfP2, PfP2CΔ40 and PfP1 proteins, against a panel of 7 anti-PfP2 monoclonal antibody supernatants (neat) and a rabbit α-PfP1-polyclonal antibody (1∶ 2000 dilution). **II:** Titration of polyclonal anti-PfP1 antisera against 100 ng PfP1 and PfP2 proteins. **III:** ELISA of 7 different PfP2 peptides (details described in Methods section) to map the epitope of E2G12. It binds to peptide 5, amino acid 27 to 49 (H-NVLGA VNADV EDEVL NNFID SLK-OH). (**C**) About 4 µg each of recombinant proteins-rPfP2, rPfP2CΔ20, rPfP2CΔ40, GST-PfP1, GST-PfP0C and GST-were resolved on 12% SDS-PAGE. (a) Coomassie stain of the gel; (b), (c) and (d) are immunoblots probed with (b) α-P0 mAb E5F4 [36]; (c) α-P2 mAb A12D9; and (d) α-P2 mAb E2G12. (**D**) Immunoblots of 80 µg of *P. falciparum*, *P. berghei* and *P. yoelii* total parasite lysates probed with (I) α-P2 mAb E2G12 and α-P0 mAb E5F4; (II) Immunoblots of gradient SDS-PAGE of *P. falciparum* parasite crude protein, probed with α-P2 mAb E2G12 and α-PfP1-polyclonal antibodies; (III) Immunoblots of 50 µg each of *P. falciparum*, *Toxoplasma gondii*, mouse and human protein extracts, probed with E2G12. Actin was used as a loading control.

Full-length *P. falciparum* P2 protein (PfP2), and certain carboxy-terminal deletion constructs, (P2Cdel20 and P2Cdel40), were expressed as His-tag fusion proteins, and several monoclonal antibodies (mAbs) were generated against these (Figure 1A, B). Full length *P. falciparum* P1 protein (PfP1) was expressed as a GST-fusion protein. GST-PfP0C, which contained 80% of C-terminal PfP0 protein [28], and the PfP0-specific mAb E5F4 [39] were also used in this study. Amongst the panel of monoclonals generated against the PfP2 protein, two classes of mAbs were detected; the E2G12 type and the A12D9 type (Figure 1BI). The A12D9 type mapped against the carboxy-terminal domain and cross-reacted with PfP1; while E2G12 set did not react with PfP1 (Figure 1BI). The polyclonal antibody raised against PfP1 did not show cross-reactivity against PfP2 under native condition (Figure 1BII). Anti-P2 mAb E2G12 was specific for *Plasmodium* P2 protein and reacted specifically with the 27–49 amino-acids (aa) peptide (Figure 1BIII) while A12D9 mapped to the 92–112 amino acids extreme-C-terminal region of P2 (Figure 1BI, Cc). All recombinant PfP2 proteins, the full length as well as the carboxy-terminal deletions, exhibited distinct higher oligomers that were SDS-resistant, while the recombinant PfP1 or PfP0 proteins did not exhibit any oligomerization (Figure 1C). In the crude *Plasmodium* protein extracts, resolved on SDS-PAGE, a protein band at about 65 kDa was noted in addition to the 16 kDa PfP2 monomer (Figure 1DI). The mAb E2G12 was found to be specific for *Plasmodium* P2 protein, and did not react with either recombinant PfP1 or the P1 protein in the parasite extract (Figure 1 B, C, D). Although the rabbit polyclonal anti-PfP1 antibody reacted with both PfP1 and PfP2 in denatured conditions (Figure 1DII), it did not react to recombinant PfP2 under native conditions (Figure 1BII). The anti-PfP1 antibodies also showed features distinct from anti-PfP2 in sub-cellular localizations evaluated through immunofluorescence assays (see below) further indicating the specificity of the anti-PfP1 antibody under native conditions. Since the extreme-C-terminal regions of PfP2 shows extensive homology with PfP1 (Figure S1B), it was natural that A12D9, which mapped to the 92–112 amino acids extreme-C-terminal region of P2, also reacted to PfP1 (Figure 1 BI, Cc). Across all *Plasmodium* species, the P2 protein is 75% identical in amino acid sequence (Figure S1A), and this explains the observation that the mAb E2G12 reacted with the rodent malaria parasites *P. berghei* and *P. yoelii* P2 proteins (Figure 1DI). The SDS-resistant protein band reactivity at 65 kDa were detected in these *Plasmodium* species as well (Figure 1DI), and upon analysis by mass-spectrometer these appeared to be oligomers of PfP2 protein (see Section on oligomerization below). It should be noted that P2, but not P0/P1, has been reported as a detergent-resistant membrane protein in a large-scale analysis of *Plasmodium* membrane proteins [40]. The mAb E2G12 did not react with *Toxoplasma gondii*, a closely related Apicomplexan parasite, or with human and mouse P2 proteins (Figure 1DIII). Overall, anti-P2 mAb E2G12 seemed to be absolutely specific for *Plasmodium* P2 protein.

**Figure 2:**
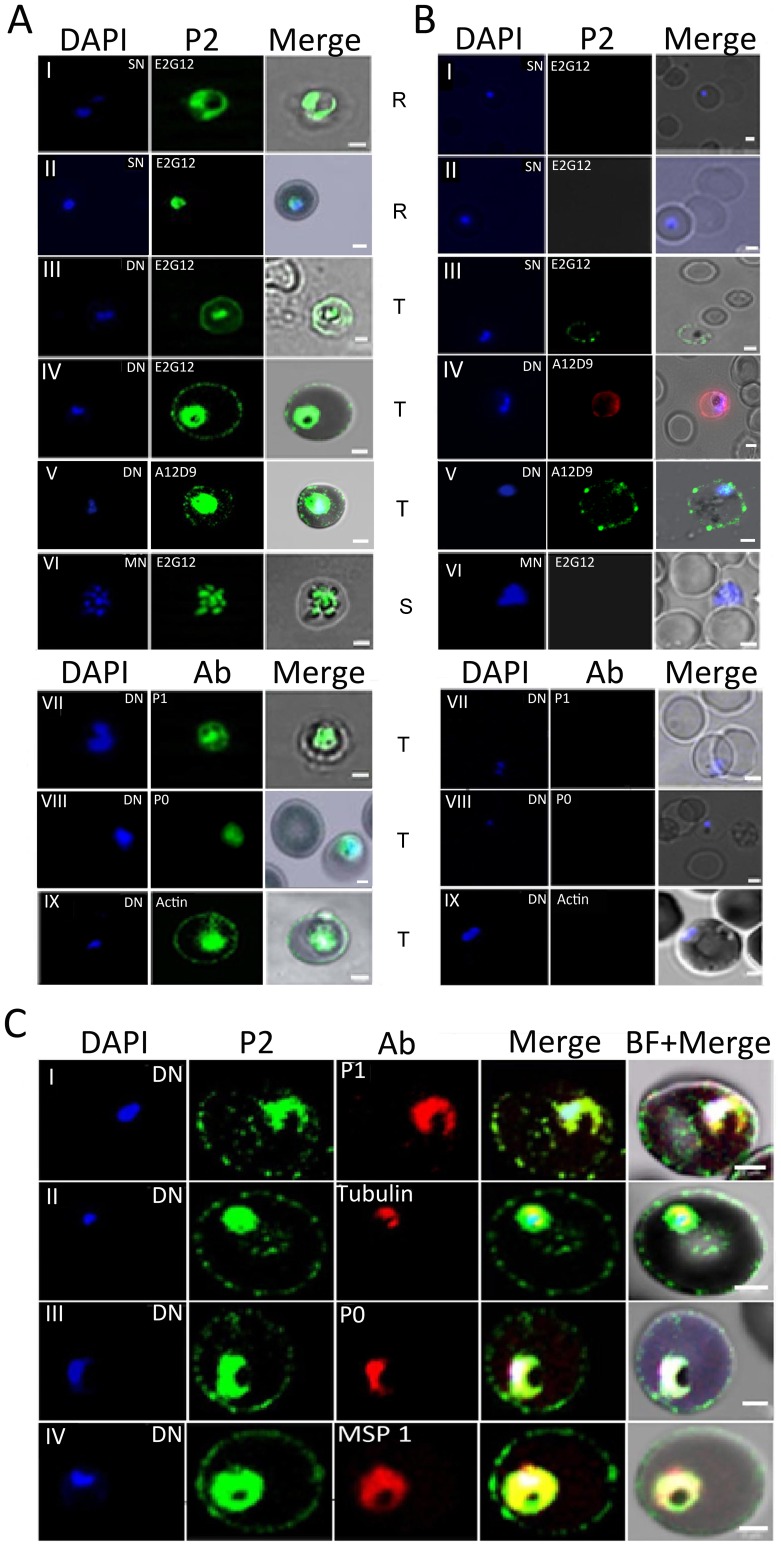
Immunofluorescence assay of *P. falciparum* infected erythrocytes (IE) using various antibodies. (**A**) & (**B**) show confocal images of permiabilized (IFA) and unpermeabilized in solution (SIFA) assays respectively, of different IE stages (I, II: Ring; III, IV, V, VII, VIII, IX: trophozoite; VI: schizont) probed with I-VI: anti-PfP2 mAbs. In all cases mAbs E2G12 was used except panels A V and B IV, V where A12D9 was used. VII: antiPfP1 polyclonal; VIII: anti-PfP0 mAbs E5F4; IX: Phalloidin (for staining β-actin). In each panel the IE nucleus/nuclei were stained using DAPI (first column), antibody staining (second column), and a merge of antibody with bright field (third column). (**C**) Localization of DAPI (blue), P2 (green); various antibodies (anti-PfP1, anti β-tubulin, anti-PfP0, and anti-MSP1) in red at the di-nuclear (DN) stage. R: Ring; T: Trophozoite; S: Schizont; SN: single nucleus; DN: Di-nuclear; MN: Multi-nuclear stages of *Plasmodium falciparum* infected erythrocytes. Scale bar indicates 2 µm.

An IFA analysis of permeabilized IEs from asynchronous cultures of *P. falciparum* 3D7 showed a transient presence of P2 protein on the IE-surface at certain stages of development (Figure 2A). This was often coincident with a dumbbell shape, elongated or crescent shaped DAPI stained nuclei (henceforth designated as the di-nuclear or DN-stage). It was not seen with the tightly packed single nuclear (SN) rings, nor the multi-nuclear (MN) schizont stage IE-surfaces (Figure 2A). Cytoplasmic P2 staining was observed at all stages (Figure 2A). To test for surface exposure, unpermeabilized solution immunofluoroscence assay (SIFA) was carried out which confirmed the presence of P2 on the outer IE surface at the DN stage but not at the SN or MN stages (Figure 2B). F-actin, present in the erythrocyte just under the membrane-bilayer, was not stained, demonstrating that IE was not permeabilized under these SIFA conditions (Figure 2BIX). Two different anti-P2 mAbs, E2G12 and A12D9, as well as two different secondary antibodies with different flurophores (Alexa 488 and 594) showed similar results, confirming the specific surface reactivity of PfP2 protein (Figure 2 A,B). Several antibodies against other proteins of *Plasmodium* such as PfP1, β tubulin, PfP0 and merozoite surface protein 1(MSP1) were tested, and none of these proteins exhibited IE surface localization (Figure 2C).

### Oligomerization of PfP2 protein correlates with P2 localization on IE surface

**Figure 3:**
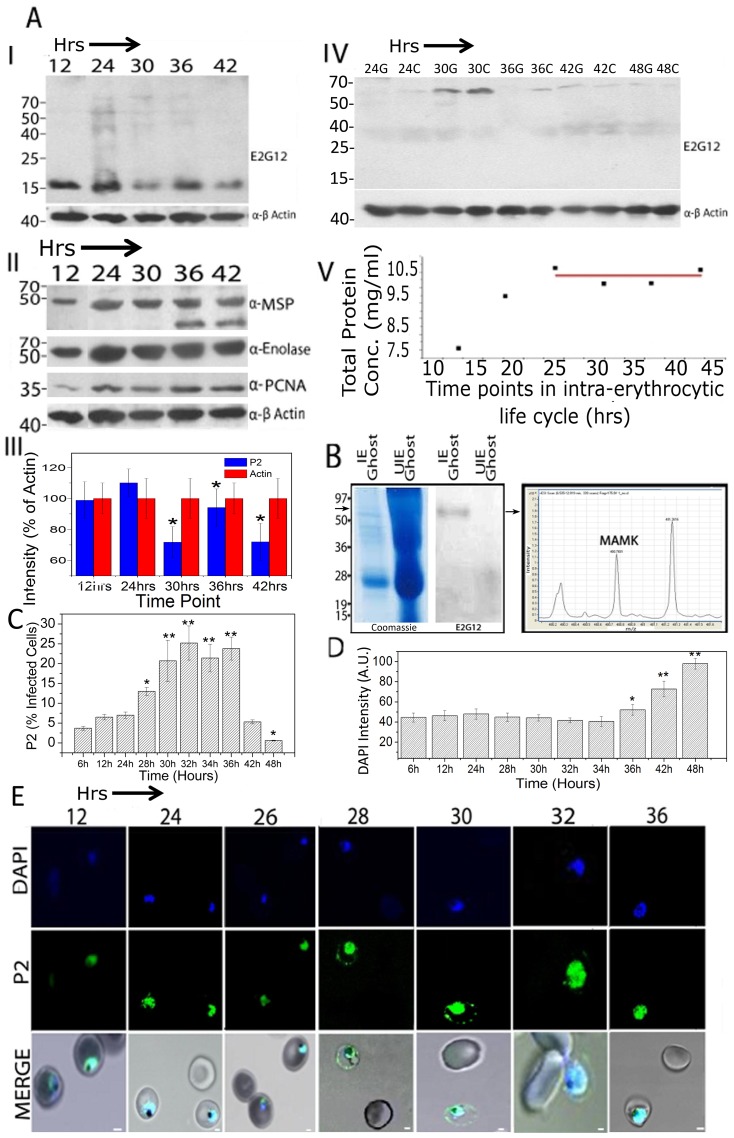
Immunoblot, mass spectrometry, flow cytometry and IFA of parasite and RBC localized PfP2 protein. Infected erythrocytes were lysed gently with saponin to separate parasite and the IE components. **A:** Synchronized *P. falciparum* parasites were harvested at various time points post-merozoite invasion (PMI) during the development of parasite in erythrocytes. Immunoblots with 40 µg of total parasite protein preparation (I and II) and 80 µg of IE ghost and IE cytosol (IV) were probed with various antibodies. IE ghost and cytosol at various time points are depicted as G and C lanes, respectively. Panel III shows a histogram of fluctuation of parasite P2 protein intensity averaged over three independent experiments, normalized to β-actin levels. Panel V shows the total parasite protein concentration at each time point PMI. **B:** Coomassie stain and immunoblot of infected erythrocyte (IE) ghost (40 µg) and uninfected erythrocyte (UIE) ghosts (120 µg) resolved on SDS-PAGE, probed with E2G12. The arrow in the Coomassie stained gel (65 kDa region) was cut out and used for Mass spectrometric analysis using Electronspray Ionization Mass spectrometry/Mass spectrometry (ESI/MS/MS) which yielded the peptide MAMK of P2 protein (see Table S1). **C:** Flow cytometric data on population of cells positive for IE surface exposed P2 using E2G12 across various stages of *P. falciparum*. **D:** Flow cytometric analysis of IEs using DAPI across the 48 hrs life cycle of *P. falciparum*. For analysis, total 6 million cells were counted. The statistical comparison for each data point is made with the corresponding control (no antibody) value. * *P*<0.05, ** *P*<0.01. **E**: IFA of synchronized *P. falciparum* cells using DAPI (blue), P2 (green), and bright field of IE at various time points PMI in parasite development. Scale bar indicates 2 µm.

Both the recombinant PfP2 and the asynchronous parasite P2 proteins in the *Plasmodium* cell extracts showed a propensity to form SDS-resistant P2-oligomers (Figure 1 Cd,DI; S2). The infected erythrocytes were treated with mild saponin and very carefully separated into IE ghost, IE cytosol and parasite protein preparations (see Methods Section). The immunoblots, of such parasite protein preparations obtained from asynchronous (Figure S2) and different stages of synchronized *P. falciparum* cultures, were probed using anti-PfP2 mAb E2G12 and various other antibodies (Figure 3A). The results yielded several interesting features. In the parasite extract, apart from the PfP2 monomer, SDS-resistant oligomeric species started to appear at 24 hrs post-merozoite invasion (PMI) stage, the concentrations of which reduced considerably by 42 hrs PMI (Figure 3AI). Also there appeared to be oscillations in the PfP2 monomer concentrations (Figure 3AI). We did not detect such oscillatory changes with other parasite proteins such as MSP1, Pf-enolase, or β-actin (Figure 3AII). As expected, *Plasmodium* proliferating cell nuclear antigen (Pf PCNA) concentration was lower prior to 24 hrs, but even that did not vary significantly after 24 hrs PMI (Figure 3AII). The ratio of concentrations of parasite PfP2 monomer and β-actin averaged over three independent experiments is shown in Figure 3AIII, and significant concentration differences were found between two consecutive 6 hr time points from 30–42 hrs PMI. The total protein content of the parasites did not vary significantly between 24 to 42 hrs PMI, indicating that total increase in protein synthesis required for subsequent cell-division/schizogony in IE was by and large over by 24 hrs PMI (Figure 3 AV). Why the PfP2 monomer undergoes oscillations is not apparent, but it is suggestive of some regulation on the total PfP2 monomeric protein quantity within the cell. The oligomerization of PfP2 protein did not appear to be dictated by the total PfP2 monomer concentration, since 36 hrs PMI parasites had a higher concentration of PfP2 monomer but showed lower quantities of oligomers. It was noteworthy that no monomeric PfP2 was seen in the erythrocytic ghost and the RBC cytosol, but mainly the 65 kDa SDS-resistant protein band was detected, predominantly at 30 hrs PMI (Figure 3AIV). The intensity of the bands appears to be comparable, or even higher, in the RBC cytosol fraction versus IE ghost at several time points. Although we do see some staining of PfP2in the RBC cytosol in IFA (Figure 2), the surface staining is always greater than the RBC cytosol staining. The higher proportion in the biochemical data is likely to be due to some degree of solubilization of PfP2 from the IE ghost fraction during processing.

To test whether there was any reactivity of anti-P2 mAb with uninfected RBC ghost, immunoblot analysis was performed using infected and uninfected erythrocyte ghosts, followed by Electronspray Ionization Mass spectrometry/Mass spectrometry (ESI MS/MS) analysis (Figure 3B). The IE-ghost showed the presence of the 65 kDa band, but no monomer reactivity; while uninfected RBC ghost showed no reactivity with anti-P2 mAb E2G12, despite 3-fold excess protein loading (Figure 3B). The 65 kDa band cut out from the IE ghost lane yielded MAMK peptide of P2 upon mass spectrometry (MS) analysis (Figure 3B). IE ghost of parasites at 30 hrs PMI and asynchronous *P. falciparum* parasite extracts were immunoprecipitated using E2G12, separated on SDS-PAGE and the 65 kDa band was subjected to MS (Tables S1 and S2; Figure S3 A,B). The peptide NVLGAVNADVEDEVLNNFIDSLK from PfP2 was detected in both the preparations. In the parasite protein the score for this peptide score was 475 while in the IE-ghost the peptide score was 51 with a coverage of 47.5% (Tables S1 and S2). Other IE-related *Plasmodium* proteins such as PfEMP1, STEVOR and RIFIN peptides were also detected in the IE-ghost preparation with a low score (Table S1). The MAMK motif is conserved in all species of *Plasmodium* P2 protein (Figure S1A), and a *Plasmodium* data base (www.Plasmodb.org) search showed that MAMK motif is not present in any other *Plasmodium* protein, nor in the P2 protein of any other species. The 65 kDa portion of the uninfected erythrocyte ghost did not show any signature of PfP2. A representative spectrum for the NVLGAVNADVEDEVLNNFIDSLK peptide obtained from the parasite is shown in Figure S3 B. Another peptide VLNNNGLGSSMNSYNEAYKK, from a hypothetical protein of *P. falciparum*, was also seen consistently at the 65 kDa position from the parasite extract IP, albeit with a low score of 17 (Table S2). This peptide maps to a gene (PFD0985w), recently annotated as transcription factor with AP2 domain(s), with a putative molecular weight of 400 kDa (www.Plasmodb.org). It is not clear as to whether PfP2 forms a complex with this protein. FPLC of asynchronous parasite extracts show large complexes containing PfP2 eluting at void volume (>600 kDa) (Das and Sharma, unpublished data). However, further analysis is needed to elucidate the role of this protein (if at all) in the physiology of PfP2. Since the score of all other peptides at 65 kDa was low, and the mass of this SDS-resistant P2 molecular species fitted a homotetramer, it appears that the 65 kDa protein band largely consists of an SDS-resistant 65 kDa PfP2-homotetramer. The homotetramer was also observed in the IE-ghosts from the two rodent parasite species *P. berghei* and *P. yoelii* (Figure S3C). In the RBC cytosol there was no contamination of alpha-spectrin (Figure S3 CIII), but RBC cytosol always contained more PfP2 protein compared to IE ghost. Since IFA data does not support this observation, the data suggests a peripheral nature of attachment of PfP2 to IE membrane, which is perhaps easily solubilized during RBC ghost and cytosol preparation.

In order to determine the population of IE showing surface localization/exposure of PfP2, a flow cytometric analysis of solution stained cells was carried out and the fraction of cells, showing staining beyond a cut-off set up on flow cytometer (Figure S4), was measured (Figure 3C). Significant number of IE started showing staining at 28 hrs PMI and at the peak about 25% of cells showed antibody staining between 32 to 36 hrs PMI. There was a rapid decline in anti-P2 staining at 42 hrs PMI and beyond. As expected, the DAPI mean fluorescent intensity started showing significant increase post 36 hrs PMI (Figure 3D). In terms of intensity of surface P2 staining, confocal microscopic observations of stage specific IFA showed the presence of IE-membrane localized P2 at highest intensity during 28–30 hrs PMI (Figure 3E). Although only 25% IE score as P2-surface positive on flow cytometric analysis, on the confocal microscope at least 90% of 30 to 36 hrs PMI cells show P2 surface staining on SIFA. This difference is likely to be due to weak staining of IE-exposed PfP2, and the differential sensitivity of the two methods.

These results document that the total amount of protein PfP2 in the parasite, and the processes of oligomerization of PfP2 and its transport to the IE-membrane surface as an SDS-resistant homotetramer, are regulated in a development specific manner. The SDS-resistant P2-oligomerization occurs in the parasite at 24 hrs, just prior to the IE-membrane localization of P2 protein at 30 hrs PMI. The window of IE surface exposure of PfP2 is mainly from 28 to 36 hrs PMI, and the SDS-resistant 65 kDa PfP2 protein, occurring largely in the IE ghost and cytosol, is most likely to be a P2 homotetramer.

### PfP2-GFP expression indicates translocation of endogenous ribosomal P2 protein to the IE surface

**Figure 4:**
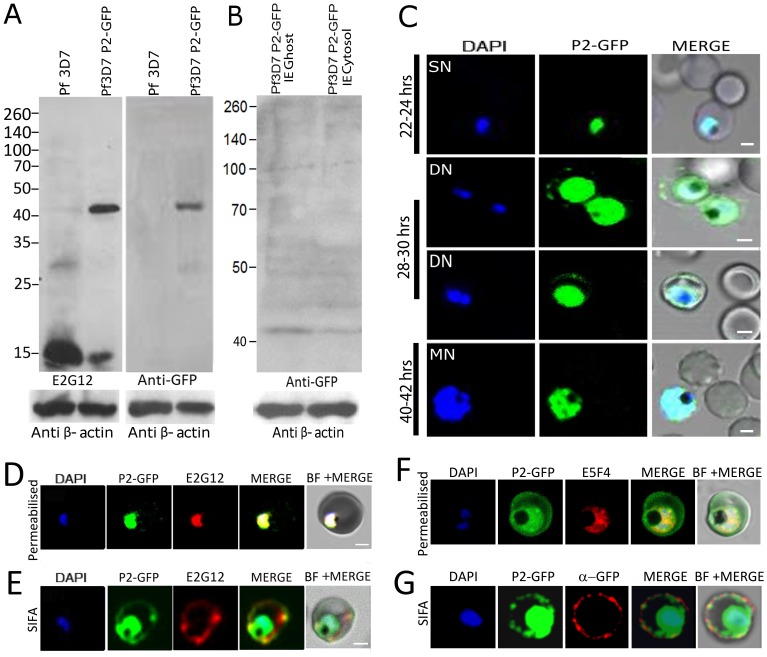
P2 expression in transiently transfected P2-GFP *P. falciparum* strain. (**A**) Immunoblot of 40 µg total protein extract at 24 hrs PMI from Pf3D7 and Pf3D7 P2-GFP strains, probed with anti-P2 mAb E2G12 and anti-GFP antibody. (**B**) Immunoblot of 40 µg each of proteins from Pf3D7 P2-GFP infected RBC ghost and cytosol at 30 hrs PMI separated on a 12% SDS-PAGE, probed with anti-GFP antibody. (**C**) Confocal microscopy of P2-GFP transfected cells at different stages of parasite growth. DAPI (blue), P2-GFP (green) (**D,F**) Confocal microscopy of permeabilized IE showing D) Red: anti-PfP2 mAb E2G12; F) Red: anti-PfP0 mAb E5F4; Green: P2-GFP staining of Pf3D7 P2-GFP infected RBCs. (**E,G**) Solution immunofluorescence (SIFA) of DN Pf3D7 P2-GFP infected RBCs showing E) Red: anti-PfP2 mAb E2G12; G) Red: anti-GFP antibody; Green: P2-GFP staining of Pf3D7 P2-GFP infected RBCs. In all images the nuclei of the parasite were stained blue by DAPI. Scale bar indicates 2 µm.

In order to test whether the endogenous ribosomal PfP2 protein translocates to the IE surface, *P. falciparum* Pf3D7 strain was episomally transfected with P2-GFP construct using the constitutive *hsp86* gene promoter region (Figure S5). This would result in a fusion protein of P2 with the green fluorescent protein (GFP) at its C-terminal end. Western blot of Pf3D7 and Pf3D7P2-GFP parasite crude protein showed the reactivity of PfP2-GFP at the expected 42 kDa band, using both anti-P2-mAb E2G12 and anti-GFP antibody (Figure 4A). In the Pf3D7P2-GFP transgenic line, the expression level of native P2 was found to be greatly reduced as compared to the 3D7 strain, indicating an influence of GFP-P2 on the total amount of endogenous P2 protein (Figure 4A). While the higher P2-oligomers were seen in 3D7, these were not observed in the transfected line, either in the parasite or in the IE-ghost and cytosol (Figure 4A, B). Faint bands were detected at the monomeric level at 42 kDa position (Figure 4B). It is possible that the oligomer of P2-GFP was unstable and got degraded. Since P2 is merely a 16 kDa protein, to which a 26 kDa GFP protein has been fused, it is not surprising that the oligomerization is compromised in the fusion protein. The use of smaller tags on P2 may help in generating more stable and efficient tagged-P2 oligomers.

Confocal microscopy of PfP2-GFP transgenic line showed the presence of GFP on the IE-surface at 28–30 hrs PMI trophozoite stage, but not at 22–24 hrs or 40–42 hrs PMI (Figure 4C). The surface localization of GFP was of low intensity, and could be detected only on about 50–60% of this stage of parasites with an overexposure for GFP. The parasite internal cytoplasmic expression of P2-GFP was very robust (Figure 4C–E). At the single or multinucleated stages, no GFP fluroscence was detected on the RBC surfaces (Figure 4C). Using permeabilized IFA, cytoplasmic staining of anti-P2-mAb E2G12 co-localized with the GFP fluorescence in transfected cells (Figure 4D). This showed that the distributions of endogenous PfP2 and the GFP-fusion P2 protein were similar, indicating normal spatio-temporal expression of the ectopic PfP2-GFP fusion protein. Unpermeabilized SIFA at the DN stage also showed that the P2 staining and GFP co-localized on the IE surface (Figure 4E). IFA carried out with anti-PfP0 mAb E5F4 showed that PfP0 was completely internal to the parasite, while the GFP can be seen within the parasite, in the RBC cytosol as well as localized on the IE surface (Figure 4F). Unpermeabilized SIFA at the DN stage, using anti-GFP antibody, also showed that the antibody staining and GFP co-localized on the IE surface (Figure 4G). The presence of P2-GFP protein on IE surface at the requisite parasite developmental stage (28–30 hrs PMI, or at a distinct DN state) strengthens the conclusion that endogenous P2 molecule moves to the IE surface at this stage. We note the absence of multimers in the transfected parasites at 24 hrs, and that of tetramers in the IE ghost/cytosol at 30 hrs PMI (Figure 4A, B), and actually observe monomeric GFP-fusion proteins on IE ghost/cytosol (Figure 4B). It is possible that the tetrameric fusion protein is unstable and we are unable to detect it on immunoblots of IE ghost/cytosol preparations. It is also possible that although oligomerization of PfP2 precedes the translocation of PfP2 to IE membrane, oligomerization is not mandatory for the surface expression of PfP2.

### Treatment with anti-PfP2 mAbs cause reversible arrest of *P. falciparum* infected erythrocytes at the first nuclear division

**Figure 5:**
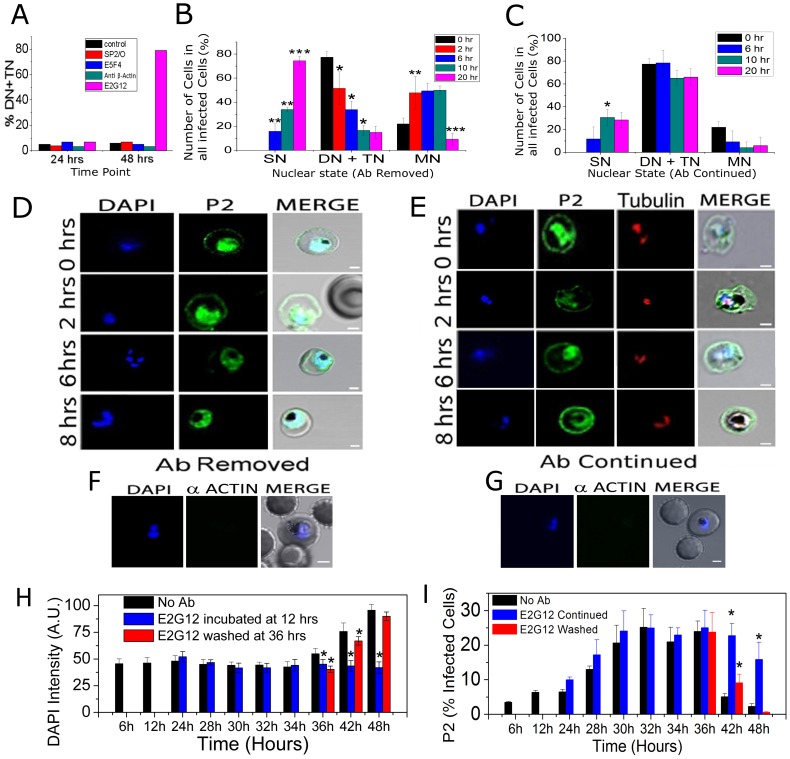
Effect of anti-P2 mAb E2G12 on *Plasmodium* cells. Synchronized *P. falciparum* cells were treated with no antibody (control); SP2/O: medium from a non-Ig-secreting cell line; and various mAbs (anti-PfP0: E5F4; anti-β actin and anti-PfP2: E2G12) each of the same isotype IgG1, used at 1 mg/ml final concentration. The distribution of the SN/DN/TN/MN stages of the IEs were determined through DAPI count. SN: IE with single nucleus; DN: IE with di-nuclei; TN: IE with tri-nuclei; MN: IE with >3 nuclei (**A**) Various antibodies were added at 12 hrs PMI and the population was scored at 24 and 48 hrs. (**B–C**) Synchronized *P. falciparum* cells were treated with E2G12 mAb for 24 hrs starting from 12 to 36 hrs PMI. At 36 hrs the arrested cells were washed and split into two flasks and cultured for further 20 hrs with and without E2G12 (antibody continued and removed, respectively). The population of IE were scored using DAPI at 0, 2, 6, 10, 20 hrs post washing; corresponding to 36, 38, 42, 46 and 56 hrs PMI. *P<0.05, **P<0.01, ***P<0.001. Significance of each time point was calculated with respect to the immediate previous time point; n = 5. Standard deviation has been represented as an error bar. (**D, E**) Permiabilized IFA of cells at 0, 2, 6 and 8 hrs post washing, corresponding to 36, 38, 42 and 44 hrs PMI using DAPI and anti-P2 E2G12 mAb. (D): after E2G12 mAb was washed off and (E) with E2G12 mAb treatment continued. Anti-tubulin antibodies were also used to stain the microtubule organization center (MTOC) of the arrested cells. (**F, G**) SIFA for actin staining of cells that were treated for 24 hrs of E2G12, and then with E2G12 washed off (F) and E2G12 continued (G). No actin staining was observed showing that treatment with E2G12 did not cause membrane permeabilization. Scale bar indicates 2 µm. (**H, I**) Flow cytometric analysis of DAPI and P2 staining of E2G12 treated cells (E2G12 added at 12 hrs PMI) under SIFA conditions. Staining was done in control cells (no antibody), and in cells with E2G12 continued and after E2G12 was washed at 36 hrs PMI. For analysis, total 6 million cells were counted. Representative data for the dot-plot and the P2 fluorescence intensity data are shown in Figures S8 and S9, The intensity of DAPI values shown in panel H are in comparison with the unstained cells. The *p* value depicts a statistical comparison with the control (no antibody) value for each time point. * *P*<0.05, n = 3.

Synchronized *P. falciparum* culture, treated with anti-PfP2 mAb E2G12, showed no visible effect up to 24 hrs, but about 80–85% cells were found to be arrested at mainly the di- and some tri-nuclear (DN and TN) stages at 48 hrs (Figure 5A). Each of the control mAbs, such as anti-PfP0 E5F4, anti-β-actin (both of the same isotype IgG1 as E2G12) and SP2/O (a non-Ig-secreting cell line used for the generation of E2G12) culture supernatant concentrated the same way as the E2G12 supernatant, were used at 1 mg/ml final concentration. None of these controls exhibited such an arrest (Figure 5A). Anti-PfP2 mAb A12D9 showed results very similar to E2G12 (Figure S6). Such an effect of the mAbs was observed at concentrations of 1 mg/ml, after a prolonged incubation with the treatment starting at 12 hrs PMI (Figure 5A, S6). However, even at a comparatively lower concentration of 20 µg/ml, about 60% cells could be arrested with 6 hrs of incubation. The maximum effect on nuclear division arrest was seen with treatment from 24 to 36 hrs PMI, and there was no arrest if incubated between 0–18 hrs or post-42 hrs PMI.

We noted that we did not see 100% IE arrested but typically saw about 75–80% arrested at the DN/TN stage (Figure 5A–C, S6 A). The remaining cells were at the MN stage and this fraction decreased slowly with time even with E2G12 continued (Figure 5 C), indicating that these cells escaped the arrest and progressed through. An analysis of the total number of parasitized cells using Giemsa stain indicated that anti-P2 treated IEs decreased significantly by 48 and 60 hrs (Figure S7 A). This was possibly because of the increase in parasitemia in the control culture (170% at 48 hrs and 220% at 60 hrs PMI; Figure S7 A) due to second round of parasite invasion. In order to check the effect of E2G12 on total parasitemia within the 48 hr development of IE, we performed flow cytometric analysis of DAPI positive cells, with the addition of E2G12 at 12 hrs PMI (Figure S7 B). It was observed that parasitemia decreased to about 70% by 48 hrs PMI, and that the selective lysis of E2G12 coated cells was limited to 20–30% infected cells (Figure S7 B). It was noted that there was a trend towards decrease in parasitemia right from 24 hrs onwards, concomitant with P2-surface exposure (Figure S7 B).

To test whether this arrest could be reversed, IEs were incubated from 12 to 36 hrs PMI with anti-PfP2 mAbs, and then the cultures were washed thoroughly. These cells were then subjected to further culturing with and without the presence of antibodies. Upon removal of the mAbs the arrest was reversed and cells with greater than 3 nuclei (TN-stage) were observed rapidly (Figure 5B). A large fraction of the cells resumed nuclear division within 2 hrs, and more than 50% cells showed more than 3 nuclei within next 2–4 hrs (Figure 5BD, S6 B), whereas in the continued presence of anti-P2 mAb the cells remained stuck at the DN stage (Figure S6 C), and the DN/TN fraction remained unchanged (Figure 5C). Permeabilized IFA using anti-P2 mAb E2G12 and anti-tubulin antibodies showed that the arrested DN cells exhibited surface-P2 staining and were always at a state when two centriolar plaques were separated (Figure 5E). *P. falciparum* infected cells were incubated with E2G12 from 12 to 36 hrs PMI, washed and then these IE were subjected to SIFA using anti-β actin antibodies (Figure 5 F, G). No actin staining was seen, and thus, the arresting property of the anti-P2 mAbs was not due to membrane permeability upon prolonged incubation with E2G12 allowing the mAbs an internal access.

A flow cytometric analysis of the P2 surface stained cells showed that the population of cells with surface P2 was maximum at 30–36 hrs PMI in about 25% of cells, and that this population rapidly decreased significantly over the next 6 hrs to 5%, concomitant with an increase in DAPI (Figure 5 H,I). A representative flow cytometric dot-plot data of anti-P2 fluorescence versus DAPI intensity for the synchronous culture of *P. falciparum* at various stages is shown in Figure S8, and the population count of E2G12 stained cells is shown in Figure S9. A comparison of DAPI staining between control and E2G12 treated IEs at 36 hrs PMI showed that E2G12 treated cells exhibited significantly lower DAPI intensity, possibly because of the arrest at DN/TN stages (Figure S8). By 48 hrs there was virtually no PfP2 staining observed in the control cells (Figure 5I, S9 A). In the continued presence of E2G12, the population of P2 stained cells increased maximally between 30–36 hrs PMI, but remained high up to 42 hrs and then declined by 48 hrs (Figure 5I, S9 B). The decrease from about 25% to 15% in the number of P2 stained cells in E2G12 continued situation from 36 to 48 hrs (Figure 5I) could represent both the escaped cells and selectively lysed cells. The DAPI staining clearly showed the difference in nuclear content with continued E2G12 treatment versus no antibody treatment (Figure 5H, S8). With E2G12 washed off at 36 hrs PMI, the cells showed a rapid decrease in the staining of surface exposed PfP2 within 6 hrs. By 48 hrs PMI, both surface-P2 and DAPI staining of E2G12 washed cells reached levels comparable to that of control cells (Figure 5 H,I; S9).

*P. yoelii* IE also exhibited arrest upon incubation with the mAb E2G12 for 12 hrs, and could be rescued upon removal of antibodies (Figure S10 C). Upon removal of antibodies, the RBC surface localized P2 gradually disappeared and multinucleated cells were observed (Figure S10 A, B, C). However, unlike arresting at the distinct dumbbell shaped di-nuclear (DN) stage of *P. falciparum*, the *P. yoelii* cells showed a large number of crescent shaped nuclei. These were scored as single nucleated (SN) cells (Figure S10 C). Hence in *P. yoelii* both the SN and DN were predominant after incubation with E2G12 (Figure S10 C). Antibody arrested live cells were stained using Tubulin tracker and anti-P2 mAb E2G12 and once again, the presence of two tubulin plaques (green) was observed simultaneous with P2 staining (red) on the arrested infected RBC surface (Figure S10 D, upper panel). Another cell division marker, PCNA, was detected in the nucleus when P2 was present on the IE-surface (Figure S10 D, lower panel). Similar results were seen for *P. berghei*.

These results demonstrate that *P. falciparum* IEs can be reversibly arrested at the onset of first nuclear division specifically through the treatment of anti-P2 mAbs. Like *P. falciparum*, erythrocytes infected with rodent parasites could also be reversibly arrested using anti-P2 mAbs. Since P2 protein is very conserved, these results open up the possibility of targeting IE cells selectively, without affecting uninfected RBCs, for all *Plasmodium* species using anti-P2 mAbs. These results also indicate an important role of P2 protein on IE surface in *Plasmodium*. The P2 proteins of eukaryotic cells are deemed non-vital since yeast *S. cerevisaea* P2 protein can be knocked out, and such cells are viable [34]. Knocking out of the P2 protein was attempted in *P. berghei* using standard constructs. However, the P2 gene could not be knocked out despite several attempts, although simultaneous knock-outs of Pf16 and MSP7 [41], [42] could be obtained.

### Treatment with anti-PfP2-mAbs disrupts the tubovesicular network of *Plasmodium-*infected erythrocytes

**Figure 6:**
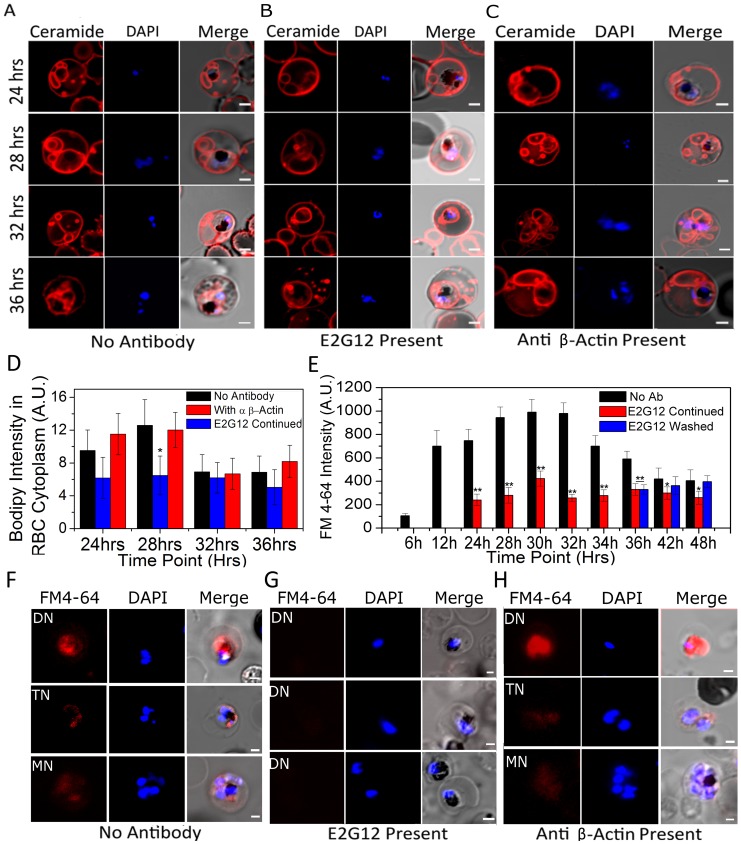
BODIPY-ceramide and FM4-64 staining of live infected erythrocytes in the presence and absence of anti-P2 mAb E2G12. (**A–C**) Control, anti-P2-mAb E2G12 and anti-β actin antibodies were added to synchronized cultures of *P. falciparum* at 12 hrs post merozoite invasion. Cells were harvested from 24 hrs to 36 hrs at various time points, washed, incubated with BODIPY-ceramide for 15 min at 37°C, and the structure of TVN [41], [42] was recorded in IEs. **D.** Quantification of BODIPY ceramide intensity in the infected RBC cytosol averaged over at least 15 infected red cells at various time points under control (no antibody), and in the presence of E2G12 and anti-β actin antibodies as described in Materials and Methods. **E–H.** Infected erythrocytes were treated with FM4-64 for 30 mins to measure the lipid import [42]; **E.** Flow cytometric analysis depicting mean fluorescence intensity (MFI) of FM4-64 uptake in infected RBCs at various time points; with no antibody (Ab), in the presence of anti-P2 mAb E2G12 and after removal of E2G12 (washed) at 36 hrs PMI. Significance (*P<0.05 and **P<0.01) of each data point was calculated with respect to the immediate previous data point. (**F–H**) Confocal microscopy of FM4-64 uptake under control (no antibody); with anti-P2 mAb E2G12 and anti-β actin treated cells at various stages of development. In all images nuclei were stained with DAPI. Scale bar indicates 2 µm.

Results obtained so far strongly indicated that anti-P2 mAbs were exerting an effect across several membranes on the nuclear division in *P. falciparum*. Therefore it was envisaged that the anti-P2 mAb treatment might be affecting some IE signaling and/or crucial nutrient uptake. It has been documented that the parasite imports several nutrients via a tubovesicular network (TVN) [43]–[45]. In particular, it has been observed that lipid intake, which is very crucial for the intra-erythrocytic growth phases [46], [47], occurs through the TVN [45]. TVN can be visualized by BODIPY-ceramide staining [43], [45] (Figure 6A, Supplementary video S1). TVN is seen in the IE in the early trophozoite stages [44], much before the P2 localizes to the IE-surface at the late trophozoite stage. It was observed that the TVN structures were similar in the IEs in the absence or presence of anti-P2 mAbs up to 24 hrs post-merozoite invasions, but by 32 and 36 hrs the TVN structures got fragmented in the presence of anti-P2 mAb E2G12 (Figure 6B, Supplementary video S2). Several control antibodies, such as anti-β actin antibodies, anti-MSP1, anti-PfP0 and anti-PCNA, were tested and none of these disrupted the TVN structures. A representative data set with E2G12 and anti-β actin antibodies is shown in Figure 6 A–C and that of A12D9 in Figure S11 A,B. Thus, treatment with anti P2-mAbs resulted in a visible morphological degradation of the TVN structures coincident with P2 exposure on the IE surface. The staining of TVN is a qualitative assay. Nevertheless, a subtractive image analysis was performed in an attempt to quantify the TVN fluorescence. The fluorescence of the parasitophorous vacuole (which can be identified through DAPI staining and hemozoin presence) was subtracted from the fluorescence of the total IE, giving us a value for the fluorescence in the RBC cytosol, which would be reflective of the TVN stain. About 15–20 cells were analyzed for each data point and the average fluorescence of this computed RBC cytosol at various stages in parasite development is shown in Figure 6D. The results showed a decrease in such TVN stain at 24 hrs, and this decrease was statistically significant at 28 hrs PMI, indicating a reduction/disruption of TVN structures. Similar TVN disruption was observed with the rodent malarial models (Figure S11 C). Removal of antibodies resulted in the regeneration of TVN in the IE cells of both *P. berghei* and *P. falciparum* within several hours (Figure S11 CIII,DIII).

To test the functionality of the TVN in the presence of anti-P2 mAbs, lipid import via TVN was evaluated through the uptake of lipid marker N-(3-triethylammoniumpropyl)-4-(6-(4-(diethylamino) phenyl) hexatrienyl) pyridinium dibromide (FM4-64) [45], and a definite impairment in the lipid import was observed only in the anti-P2 mAb treated IEs (Figure 6E–H). A flow cytometric analysis of the uptake of FM4-64 in control cells showed that this was parasite stage dependent (Figure 6E). The lipid uptake increased significantly at 12 hrs PMI and remained high until 32 hrs PMI (Figure 6E). After 32 hrs PMI, FM4-64 amount reduced rapidly in the normal IEs (Figure 6E). In the presence of E2G12, a severe 2 to 3-fold reduction in FM4-64 incorporation was observed and this impairment was reversed through the removal of the antibodies within 6 hrs, 36 to 42 hrs PMI (Figure 6E). A representative set of images are shown in Figure 6F–H. The control anti-β actin antibody did not show any impairment in FM4-64 uptake (Figure 6H). Unlike BODIPY-ceramide, we did not observe TVN structures when we monitored FM4-64 uptake (Figure 6F–H), possibly because FM4-64 is internalized efficiently by the parasites, and accumulates rapidly in the parasite cytoplasm. This is consistent with earlier observations on TVN and lipid import in *Plasmodium* [43]–[45].

## Discussion

Our results show that the acidic ribosomal protein P2 of *Plasmodium* plays a novel extra-ribosomal role through its trafficking to the infected erythrocyte surface as a SDS-resistant homo-tetramer at the point of the first nuclear division in the erythrocytic stage of the parasite. Upon treatment with a panel of anti-PfP2-specific mAbs, parasite development was arrested at the onset of nuclear division, and this arrest could be released upon removal of the antibodies. We also demonstrated that one of the downstream event(s) of such antibody-mediated arrest is the disintegration of the TVN, resulting in impairment of lipid uptake by infected erythrocytes.

### Erythrocyte surface exposure of *Plasmodium* P2 protein

The acidic ribosomal P2 protein has been studied extensively mainly in context with the ribosomal functions in eukaryotic systems [37], [48]–[50]. The three P-proteins (P0, P1 and P2) play a structural role in the composition of the eukaryotic 60S ribosomal subunit. In yeast, the P2-null mutant was viable in rich medium and no significant effects were seen in the rates of peptide bond formation [34], [50]. However, the protein synthesis and growth rates were reduced. Thus the P2 protein is not vital for the ribosomal stalk structure but contributes mainly to the efficiency of the ribosomes. Heterogeneity of P-proteins in ribosomal composition has been reported, and ribosomes deficient in P1/P2 proteins have been observed in the stationary phase of growth [48]. It is of interest that the pattern of protein expression in the absence of P1 and P2 proteins is distinct from that in the presence of these acidic proteins [34], [50]. It was shown that such a differential expression pattern was not due to translation error or termination suppression, but was postulated to be due to differential translation modulation, and/or due to extra-ribosomal properties of these proteins [34].

Ribosomal proteins are known to play varied roles besides protein synthesis [51]. We have earlier demonstrated that P0 protein plays a protective role in *Plasmodium*, since PfP0 protein was detected on parasite surface [28], [29] and anti-P0 antibodies selectively inhibited the growth of the parasites, both in culture [27], [39] and *in vivo*, through passive immunization followed by rodent parasite challenge [30]. In *Neisseria gonorrhoeae*, the functional orthologue of P2 (L12), is surface localized; and is shown to mimic human chorionic gonadotropin implicating its role in cell invasion [38]. There have been certain associations of ribosomal protein expression with cancer, but those have been subscribed to altered cellular protein synthesis [52]. Like yeast, *Plasmodium* P1/P2 proteins do not appear to be absolutely essential for ribosomal functions, since we have shown that ribosomes containing just PfP0 (without any P1/P2 proteins) were capable of synthesizing proteins in a complementation study in *Saccharomyces cerevisae* [31]. On the other hand, certain features-such as a) highly conserved, but specific to *Plasmodium* P2 protein amino acid stretches (for instance MAMK) (Figure S1); b) similar functional properties in the human *P. falciparum* and rodent malarial parasites *P. berghei* and *P. yoelii*; and c) the preliminary observations that we were unable to obtain a P2 knock-out in *P. berghei*; indicate *Plasmodium* specific important roles for the *Plasmodium* P2 protein. The trafficking of P2 protein to the infected red cell surface, during a specific window of parasite development, shows extra-ribosomal functions of this *Plasmodium* P2 protein. Surface exposure of a parasite protein on IEs would make the protein vulnerable to host-immune responses and conforming to this expectation most of the proteins exported to outer surface of IE are variant immuno-evasive malaria proteins [53]–[56]. Red cell surface exposure of an extremely conserved, apparent house-keeping protein P2 is indeed intriguing, since it would constitute a target for the host immune response. It is possible that the parasite uses such a conserved host-mimicking protein to keep the immune responses under control.

Several *P. falciparum* proteins get exported to the erythrocyte [53]–[56]. One of the most studied exported protein is PfEMP1, which is a variant surface antigen encoded by a family of approximately 60 *var* genes [53]–[56]. Although several *P. falciparum* proteins get exported to the erythrocyte, with the exception of the variant protein PfEMP1 (and perhaps RIFINS and STEVOR family proteins), most remain intra-erythrocytic [53], [55]. PfEMP1 proteins are large (>300 kDa) which do not possess a signal sequence, but requires its C-terminal transmembrane domain to traffic to the IE surface [53], [54]. A PEXEL motif, with the consensus sequence (R/K)X(V/F/M/l)X(E/Q/D), has been reported to mediate export of proteins to the IE surface [54], [56]. Movement of PfEMP1 onto the erythrocyte surface also requires a remarkable network of proteins that function at different steps of its trafficking through the host cell [53]–[55]. Several parasite proteins lacking the PEXEL motif also get transported to the erythrocyte [56], [57]. *Plasmodium* P2 protein has a putative trans-membrane domain (amino acids 64–84) but does not contain any signal sequence or PEXEL motif. The anti-P2-mAbs, E2G12 and A12D9, stain IE surface and can cause nuclear division arrest, and these map to the 27–49 amino acids-N-terminal and 92–112 amino acids-C-terminal regions of P2, respectively, suggesting that both these domains of P2 are IE-surface exposed. Exposure of both these domains would be unlikely if the 64–84 amino acids domain did act as the transmembrane domain. However, the protein P2 is present on the RBC surface as a homo-tetramer, and that may allow both N- and C- terminal domains to be accessible externally. It is also possible that P2 protein is present as a complex with other proteins, which provide the anchor to the IE-membrane. The comparative ease with which the P2 protein gets solubilized in biochemical studies, suggests that it may have a peripheral association with the IE-membrane.

Since there is no signal sequence or PEXEL motif in P2 protein, it is not clear what features within the *Plasmodium* P2 protein aid in the export. The oligomerization of the P2 protein may be involved in this process. In the parasite, P2 forms SDS-resistant oligomers in a stage dependent manner, coinciding with the RBC surface translocation (Figure 3). The exclusive presence of a stage-specific SDS-resistant homo-tetrameric P2 protein in the IE ghost and in the IE cytosolic fractions indicates that such oligomerization may be important for this translocation or its stability. The SDS-resistant presence of P2-homotetramer is consistent with the earlier observation of PfP2 as a detergent resistant membrane protein in *Plasmodium* [40]. P1/P2 heterodimers have been studied extensively in *S. cereviseae* and in mammalian cells [58]–[60]. It has been reported that P2 protein is stable and confers stability to the P1 protein in yeast [58]. Homo-oligomerization is also reported in yeast and mammals. While homodimers appear to be dominant in both yeast and mammals [58]–[61], in *Plasmodium* the SDS-resistant homotetramer is more relevant for surface functions. P2 homo-tetramer is also detected amongst yeast oligomers [60]. An analysis of the mechanism of oligomerization process in the parasite and a search for possible interactor protein(s) of the *Plasmodium* P2 protein may throw some light on the regulation of export of P2 to the IE surface. Parasite proteins lacking the PEXEL motif do get transported to the erythrocyte [56], [57], and P2 will add to such a list. GFP-tagged PfP2 does not appear to oligomerize and yet seems to make it to the IE surface. However, lower levels of oligomerization of P2-GFP fusion proteins may not be detected, and use of a smaller tag on P2 may resolve this question. In any case, there is likely to be homo- and heterologous protein interactions that allow P2 translocation across RBC, but the function at the RBC surface appears to be mediated by the detergent-resistant tetrameric P2. How the concentration of this IE-membrane localized P2 is down-regulated so rapidly at later erythrocytic stages is not clear at present.

### Possible modalities of nuclear division arrest

Very little is known about replication during *Plasmodium* schizogony. It is endomitotic, which implies that the mitotic spindle assembles within an intact nuclear envelope, and the nucleus divides by fission, resulting in two daughter genomes in separate nuclear envelope membranes [62]. In electron microscopy, intra-nuclear spindle microtubules can be seen in the nuclear membrane [63], [64], emanating from possibly the centriolar plaques (CP), which could be the *Plasmodium* equivalents of yeast spindle pole bodies [65]. In *Plasmodium* the ring and early trophozoite stages have a single interphase nucleus (G0), and the G1 phase and DNA synthesis (S-phase) occurs in late trophozoites. Within hours the single trophozoite nucleus begins to divide into two daughter nuclear bodies (the first M phase) and an asynchronous independent nuclear division ensues [1]–[5]. The initial transition into nuclear division cannot be detected by Giemsa staining and the terms ‘late trophozoite’ and ‘early schizont’ are equivocal as far as cell division is concerned. Recent microscopy results have shown improved resolution of the cell cycle and its phases during *P. falciparum* schizogonic divisions, and it was observed that the first CP duplication occurs between 24 and 26 hrs PMI [5].

The CP and mitotic spindles constitute the microtubule organizing centres (MTOCs) in *Plasmodium*. Certain structural features have been noted for the MTOC in *Plasmodium*. Recently, an Aurora-A-related kinase, PfArk1, was identified in *P. falciparum* and was shown to localize to mitotic spindle poles during schizogony [23]. PfArk1 appears to be essential for blood-stage development. Interestingly, PfArk1 localizes only to paired MTOCs, and it is only visible in a subset of the schizont nuclei which have short intervening mitotic spindles, suggesting that its function is restricted to a specific phase of nuclear mitosis, namely the separation of spindle bodies at the onset of mitotic spindle formation [23]. It has also been observed that a limited number of nuclei, possibly a maximum of four, undergo S/M cycle at a given time [23], indicating clear controlling mechanisms operating on the nuclear divisions in IE. There is also evidence that suggests that the centrin protein PfCEN3 is a component of MTOC [24]. In blood-stage *P. falciparum* parasites, PfCEN3 localizes to discrete regions of the nuclear membrane that coincide with the mitotic spindle poles [24]. However, in spite of these observations, the molecular mechanisms governing mitotic checkpoints in *Plasmodium* is poorly understood. Biochemical, genetic, and genomic studies indicate that Apicomplexan (and in particular *Plasmodium*) signaling networks for cell division may not rely on all of the components that are conserved in other eukaryotes [66].

We observed that *Plasmodium* P2 protein gets exported to the IE surface and associates with RBC membrane at about 26–28 hrs PMI (Figure 3E), at the same time as the CP duplication occurs. The effect of anti-P2 mAbs, causing a morphological effect only after 24 hrs, was expected since antibodies do not penetrate the IE surface and therefore cannot affect the cytoplasmic P2. Thus, the arrest of the *Plasmodium* nuclear division came as a surprise to us, since the antibody-complexed P2 protein would need to signal across several membranes such as the IE membrane, parasitophorous vacuolar membrane (PVM) and the parasite membrane. Finally it would need to signal across the nuclear membrane as well, since nuclear membrane appears to be intact during chromosomal division in *Plasmodium* [63], [64]. We hypothesized that perturbations in nutrient import could be one mechanism, which would have a downstream effect across all these membranes. This led us to examine the status of the tubovesicular network (TVN) in *Plasmodium*, which gets set up in early trophozoite for nutrient uptake [43]–[45]. We observed that the anti-P2 mAb treatment does result in the disintegration of the TVN at >28 hrs PMI, and causes an impairment of lipid uptake (Figure 6).

It is known that serum factors are essential for parasite growth and there have been attempts to dissect out the essential components for parasite growth in the erythrocytic cultures. Earlier reports have shown that fatty acids are mandatory for the parasite for cell division [46], [47], and that the lack of fatty acids causes an arrest at the trophozoite stage not allowing progress to multinucleated stages [46]. Malfunctioning parasite-specific tubovesicular network (TVN), that occurs through the use of a sphingolipid analog, DL-threo-1-Phenyl-2-palmitoylamino-3-morpholino-1-propanol (PPMP), also appeared to arrest cells at a similar stage [43], [45]. Drugs that prevent depolymerization of microtubules such as Taxol, Taxotere and epothilone A (EpA) also arrested cells at the same stage [67], [68]. In pulse experiments using Taxotere, when 5-hr pulse experiments were carried out and analyzed at 20 and 25 hrs PMI, the trophozoites were unable to undergo the first nuclear division and remained arrested as a non-segmented nucleus [69]. Taxol added after 40 hrs PMI showed no effect on the formation of merozoites and in new invasion [68]. Thus impairment through several avenues appears to converge on to the blockage at the first nuclear division.

All these severe perturbations, such as impairment in lipid import or microtubule dysfunction appear to halt the cells at the first nuclear division. In the case of anti-PfP2 mAb treatment, the blocked IE show a spread of nuclei, from enlarged single nucleus up to a dumbbell shaped nuclei, but rarely proceeding beyond that (Figure 5). Thus there appears to be a broad check-point at the first nuclear division in *Plasmodium* erythrocytic development, since blockage through multiple pathways appear to block at this stage, and not allow parasites to undergo further cell-division. Drugs which interfere with sphingolipid metabolism (such as PPMP); which inhibit depolymerization of microtubules (such as Taxol); which block microtubule polymerization (such as colchicine); as well as anti-P2-mAb treatment - all appear to bring the cells to halt at this stage [67]–[69, current study]. However, this work documents for the first time the role of a surface-exposed endogenous *Plasmodium* protein P2, blocking of which causes nuclear division arrest. Since PfP2 export to the IE surface occurs at a specific parasite cell division window, and anti-P2 mAb treatment arrests cell division, it is tempting to speculate that PfP2 protein, possibly in complex with other molecules, may be transported to IE surface to sense the external milieu and to signal whether to proceed, or to halt cell division until conditions are favourable.

Inhibitors such as PPMP and agents that affect microtubule functions (taxol, colchicine, EpA etc) also arrest the cells, and have been documented as promising anti-malarials [68]. Treatment with such agents result in lysis of the cells within minutes, while the effects of treatment with anti-P2 mAbs is milder, and cells can remain incubated with anti-P2 mAbs for long (>24 hrs- with a viability of 70–75% of cells). This precludes immunotherapeutic potential of anti-P2 antibodies in *Plasmodium*, especially since the presence of P2 protein on IE surface is transient, and PfP2 protein is conserved in the vertebrate host. Indeed, preliminary vaccination experiments using P2 protein demonstrates only a partial protection (Das *et al.*, unpublished data). However, coupling of anti-P2 mAbs to anti-malarials should allow selective targeting of drugs to *Plasmodium-*infected red cells, which has been difficult so far. Considering very little is known regarding the cell-cycle stages of *Plasmodium*, arrest of IEs with anti-P2 mAbs and then following them through the release would be extremely useful for determination of the transcriptomic, proteomic and metabolomic states of the parasite through the schizogonic cell division stages.

### Conclusion

The acidic ribosomal protein P2 of *Plasmodium* plays a novel extra-ribosomal role at the infected erythrocyte surface at the point of the first nuclear division in the erythrocytic stage of the parasite. So far, all *P. falciparum* IE surface-exposed proteins are documented to be variant antigens, and it has not been possible to selectively target the IEs. The conserved presence of the P2 protein in *P. falciparum* and in rodent species, and the identical behavior of anti-P2-mAb treated *Plasmodium falciparum*, *yoelii* and *berghei* IE, demonstrates that the mechanism of P2-mediated IE-surface function is fundamental to *Plasmodium* cell-division. Therefore, anti-malarials coupled to anti-P2 mAbs would allow selective targeting of drugs to *Plasmodium-*infected red cells to possibly all human *Plasmodium* species. In addition, synchronization of *Plasmodium* IEs using anti-P2 mAbs will help in further study of the peculiar schizogonic cell-cycle of *Plasmodium*. The oligomerization of P2 protein in the parasite, which is timed crucially to the start of cell division in IE, precedes the trafficking of P2 protein to the IE surface. Understanding the process of the oligomerization may elucidate some of the regulations operating on PfP2 protein transport. The major question regarding the role played by the P2 protein at the infected red cell surface remains elusive. Detergent resistant membrane domains are important components of cellular signals, and an analysis of the interactors of P2 protein in the infected RBC membrane may throw some light on the function of the infected-erythrocyte-surface localized P2 protein in *Plasmodium*.

## Materials and Methods

### Ethics statement

Tata Institute of Fundamental Research (TIFR) Animal House is registered under CPCSEA (Committee for the Purpose of Control and Supervision of Experiments on Animals), ministry of environment and forest, Govt. of India (registration no. 56/1999/CPCSEA) for breeding and experiments on animals. This study was carried out under strict accordance with the guidelines of CPCSEA, India, for the care and use of laboratory animals. The study was approved by the institutional animal ethics committee, TIFR, Mumbai (project no. TIFR/IAEC/2008-1) formulated by CPCSEA.

Human blood was collected from volunteers, after obtaining their written consents, for the in-vitro cultures of *Plasmodium falciparum*. The procedure for such collection, details of informed consent and the frequency of samples to be collected were in accordance with a detailed proposal approved by the Institutional Human Ethics Committee (IHEC) of TIFR. The IHEC of TIFR functions as per the guidelines of Indian Council of Medical Research (ICMR), Govt. of India.

### Parasite cultures

#### *P. falciparum* 3D7

Parasites were maintained in culture as described earlier [27]. Human blood, from healthy adults with B^+^ blood group, was collected in acid citrate dextrose as the anticoagulant. After removing the leukocytes, the erythrocytes were washed and suspended in complete RPMI (cRPMI with 0.5% Albumax). Asexual stages of *P. falciparum* 3D7 strain were maintained at 5% haematocrit in cRPMI at 37°C in a humidified chamber containing 5% CO_2_.

#### *P. berghei ANKA* and *P. yoelii17XL*

Infected RBCs were injected into male Swiss mice intraperitonially. Parasitemia was monitored on a daily basis and mice were sacrificed at around 10–40% parasitemia. Blood was harvested from the mice as per animal ethics protocol and used the same way as *P. falciparum* cultures for each set of experiments.

### Cloning and expression of PfP2 (PFC0400w) and PfP1 (PF11_0043) genes in pProExHTa and in pGEX-4T-3 vectors, respectively

P2 gene was PCR amplified from *Plasmodium falciparum* (3D7) genomic DNA using the following primers containing EcoRI (New England BioLabs, NEB) at the 5′ end and XhoI (NEB) at the 3′ restriction overhang, respectively.

Forward primer: 5′-CCCC***GAATTC***ATGGCTATGAAATACGTTGCTG-3′;

Reverse primer: 5′-GGGG***CTCGAG***TTAACCAAATAAGGAAAATCCTAAGTC-3′.

Both the PCR amplified P2 gene fragment and the pProExHTa vector (Lablife) DNA were digested using EcoRI and XhoI restriction enzymes at 37°C, purified and ligated at 16°C for 16 hrs using T4 DNA ligase (Roche, Germany, Cat no. 10481220001). DH5α competent cells were transformed, and positive clones were identified through plasmid purification and restriction digestion. PfP2CΔ20 and PfP2CΔ40 were also cloned in the same vector following the same methodology, excepting that the amplifications were carried out using the following reverse primers:

PfP2 CΔ20: 5′-GGG***CTCGAG***TTATTCTTTCTTATCTTCfTTTCTTAG-3′; PfP2 CΔ40: 5′-GGGG***CTCGAG***TTAACCACCTCCAATATTTTG-3′ keeping the forward primer as mentioned above.

GST-PfP1 construct was made by cloning PfP1 gene between EcoRI and XhoI sites of the pGEX-4T-3 vector (GE Healthcare, USA). PfP1, with one postulated intron, was amplified from a *P. falciparum* (3D7) cDNA library using the following primers:

Forward: 5′-CCCC***GAATTC***ATGGCATCAATTCCAGCATC-3′;

Reverse: 5′-GGGG***CTCGAG***ACCAAATAAGGAGAAACC-3′.

Cloned PfP1gene was transformed in DH5α competent cells. All the constructs have been schematically described in Figure 1A. The DNA (ORF) sequences of all the clones were confirmed by DNA sequencing. GST-PfP0C protein construct, used in this study, has been described earlier [39].

### Recombinant protein expression

All constructs were transformed in *E. coli* BL21 DE3 strain and protein expression was induced by 0.5 mM IPTG (Sigma-Aldrich, Inc, St. Louis, MO, USA, Cat. No. I6758). Recombinant PfP2 (rPfP2), rPfP2CΔ20 and rPfP2CΔ40 proteins were purified using Ni-NTA beads (Qiagen, Hilden, Cat. No. 30230), while GST-PfP1 and GST-PfP0C proteins were purified using GST-sepharose beads (GE Healthcare, Sweden, Cat. No. 17-5132-02). All the recombinant proteins were fusion proteins, with rPfP2, rPfP2CΔ20 and rPfP2CΔ40 containing additional 30 amino acids (aa) at the N-terminus, including 6-Histidine amino acids, totaling to 142, 122 and 102 aa, respectively. PfP2 was cloned in pQE and pET vectors to obtain non-fusion or a cleavable P2-recombinant protein. However, no stable expression of PfP2 protein could be achieved without these additional 30 amino acids. The native PfP2 protein (112 aa) moved on the SDS-PAGE at 16 kDa (Figure S3B), while rPfP2, rPfP2CΔ20 and rPfP2CΔ40 moved at 18 kDa, 13 kDa and 11 kDa sizes, respectively (Figure S3A). The native PfP1 protein contains 118 aa and moves at 17 kDa (Figure S3B), while the recombinant GST-PfP1 moved at 39 kDa on SDS-PAGE (Figure S3A). GSTPfP0C, containing 62–316 aa of the C-terminal end of PfP0 protein moved at 64 kDa [39].

### P2 gene knockout in *P. berghei*

The gene k/o strategy was followed as described in [70]. Briefly, the targeting vector for *PbP2* (PBANKA_040770) was constructed in pBS-DHFR in which the polylinker sites flank a *T. gondii dhfr/ts* expression cassette conveying resistance to pyrimethamine. PCR primers PbP2 FP1 (5′CCCC***GGGCCC***GATATCACAAAATTATATATTAACAC 3′), and PbP2 RP1 (5′ GGGG***AAGCTT*** GATAAGCTGCAACGTATTTCATAGCC 3′) were used to generate a 748 bp fragment 5′ upstream sequence of *PbRP2* from wild type genomic DNA, which was inserted into ApaI and HindIII restriction sites upstream of the *dhfr/ts* cassette of pBS-DHFR. A 492 bp fragment generated with primers PbP2 FP2 (5′ CCCC***GAATTC*** GAAGAAGAAGATGATTTAGGATTTTCC 3′) and PbP2 RP2 (5′GGGG***TCTAGA*** GAACAACTGTATATACAATGTTCC 3′) from the 3′ flanking region of *PbRP2* was then inserted downstream of the *dhfr/ts* cassette using EcoRI and XbaI restriction sites. The linear targeting sequence was released from the plasmid using ApaI/XbaI and transfected into schizont following the protocol [70]. The procedure was repeated at least three times but no drug selected parasites were obtained. For each of the three transfections where PbP2 was used, PF16 [41] and MSP7 K/o [42] was used as a control. In none of these three attempts was PbP2 K/o obtained in second pressure after transfection.

### Generation of antibodies against the P-proteins

All the monoclonal antibodies were generated (Biokolone, Chennai, India) using rPfP2 and screened against various constructs (Fig. 1). Polyclonal rabbit antibodies were raised using GSTPfP1 (Biokolone, Chennai, India). The generation of mAb E5F4 has been described earlier [39].

### Transient transfection of P2-GFP and ACP-GFP constructs in *P. falciparum* 3D7

To generate transgenic *P. falciparum* lines expressing PfP2-GFP chimeric protein, we used a modified plasmid vector pSSPF2/ACP-GFP using *hsp86* promoter sequence (Figure S5) (a kind gift from Dr. Shigeharu Sato; through Dr. P. Malhotra). We used the pSSPF2/ACP-GFP constuct [71] as a control for transfection. This plasmid vector was modified through the introduction of an A*vr*II restriction site so as to allow cutting out of the *PfACP* gene and replacing it with *PfP2* gene. *PfP2* gene was amplified from the genomic DNA by PCR using the forward primer 5′ CCCCAGATCTATGGCTATGAAATACGTTGCTG-3′ and reverse primer 5′ GGGGCCTAGGACCAAATAAGGAAAATCCTAAGTC-3′. The amplified fragment of PfP2 gene was digested with B*gl*II and A*vr*II restriction enzymes and cloned upstream of GFP to give pSSPF2/PfP2-GFP. The sequence of the clone was confirmed by an automated sequencer. Transfections were carried out as follows. Infected RBC at 0.5% parasitaemia, mainly at the ring stages, were suspended in three volumes of cytomix (BioRad) and 50 µg of plasmid DNA (prepared using QIAGEN plasmid kit) was added to 400 µl of the RBC suspension. Electroporation was carried out in a 0.2 cm cuvette (BioRad) using a BioRad gene pulser (310 V, 950 µF, 24Ω). Two days after electroporation, the antifolate drug WR99210 (Sigma) was added to cultures at 5 nM final concentration to select for transfected parasites.

### Immunoblots of recombinant and parasite proteins

The soluble recombinant proteins were harvested from bacteria after IPTG induction and purified using GST-or Ni-NTA column as described earlier. To prepare *Plasmodium* samples, the IE from asynchronous and synchronous cultures were treated with 0.15% saponin in PBS for 10 min at 37°C to liberate the parasites. The parasite cell pellet was washed at least three times with PBS or until there was no visible trace of haemoglobin in the supernatant. The parasite pellets were lysed in PBS (pH 7.4) with protease inhibitor cocktail (Sigma-Aldrich, Inc, St. Louis, MO, USA, cat no. P8340), with brief sonication at 4°C. The lysate was centrifuged at 15,000 *g* at 4°C for 30 min and the supernatant was used for the immunoblots after protein estimation using Bradford reagent (Sigma). Before loading, the protein was mixed with gel loading buffer (50 mM Tris. Cl pH 6.8, 100 mM DTT/20 mM β-Mercaptoetanol, 2% SDS, 0.1% bromophenol blue, 10% glycerol) and boiled for 10 min. Samples were resolved typically on 10–12% SDS–PAGE and proteins were transferred to methanol-activated Polyvinylidene Fluoride (PVDF) membrane (Millipore) using anode buffer (25 mM Tris.Cl pH 10.4, 10% Methanol) and Trans Blot Semi Dry Transfer system (Bio-Rad, USA). Membranes were blocked with 5% non-fat skim milk powder in 1× PBS overnight and probed with specific antibodies. Primary antibody dilution was made in PBST (PBS containing 0.2% Tween-20) and incubated with the membrane for 3 hrs at RT on a rocker. Primary antibody binding was detected by appropriate secondary antibodies conjugated to horseradish peroxidase (GE Healthcare, UK, cat no. NXA931). Dilution of secondary antibody was made in PBST. After every incubation, membrane was washed with PBST at least 4–5 time and between every 5 mins interval washing buffer was changed. The immunoblots were developed using the ECL (Amersham) also by Super signal West Picochemiluminescent substrate (Thermo Scientific, USA). The antibodies used were E2G12 (1∶100), A12D9 (1∶100), α-PfP1 polyclonal (1∶2000), E5F4 (1∶100) (generated by Bioklone, Chennai, India); anti-MSP1-19(1∶1000) (from MR4, USA); anti-β tubulin antibody (1∶1000) (Sigma Aldrich, cat no. T8328); anti-PCNA (1∶2000) (a gift from Dr. Suman Dhar, JNU, Delhi, India), α-β actin antibody (1∶1000) (Sigma Aldrich, cat no. A1978).

### ELISA of recombinant P-proteins, deletion constructs of PfP2 and PfP2 peptides

In a 96 well plate (Nunc, Denmark), 200 ng of recombinant proteins and peptides in PBS were coated and incubated at 37°C for 3–4 hrs. Wells were blocked using 5% skimmed milk in PBS for 1 hr at 37°C. Primary antibodies, anti-PfP2 mAbs (E2G12, A12D9) were used in 1∶100 dilutions whereas anti-PfP1 polyclonal antibody was used in 1∶2000 dilutions. Primary antibody was incubated for 3–4 hrs at 37°C. Anti-mouse HRP conjugated (GE Healthcare, UK, cat no. NXA931) and anti-rabbit HRP conjugated (GE Healthcare, UK, cat no. NA934) secondary antibodies were used in 1∶5000 dilutions in PBS. After every incubation, plates were washed with PBS containing Tween-20 (0.05%) for at least 5–6 times with a gap of 5–7 min between each wash. Bound secondary antibody was developed using the substrate ABTS (Roche, cat no. 10102946001) and H_2_O_2_. The OD was measured at 405 nm and plotted after the subtraction of negative control value. For each antigen, experiments were done in triplicate. For peptide ELISA, 7 different peptides along P2 protein were generated (Mimotope, Australia). These peptides were 1. PfP2 aa 41–70 (H-LNNFI DSLKG KSCHE LITDG LKKLQ NIGGG-OH), 2. PfP2 aa 67–89 (H-IGGGV AAAPA GAAAV ETAEA KKE-OH), 3. PfP2 aa 1–11 (H-MAMKY VAAYL M-OH), 4. PfP2 aa 1–20 (H-MAMKY VAAYL MCVLG GNENP-OH), 5. PfP2 aa 27–49 (H-NVLGA VNADV EDEVL NNFID SLK-OH), 6. PfP2 aa 86–101 (H-AKKED KKEEK KEEEEE-OH), 7.PfP2 aa 15–30 (H-GGNEN PSTKE VKNVL G-OH).

### Solution Immunoflurescence Assay (SIFA) and permeabilized Immunofluoroscence Assay (IFA)

SIFA of *P. falciparum*, *P. berghei* and *P. yoelii* infected RBC population was performed in solution. Infected blood was centrifuged at 500 *g* for 5 min, pelleted RBCs were washed twice, and resuspended in complete-RPMI (cRPMI) medium. Cells were fixed using 0.25% glutaraldehyde in PBS for 20 min at 4°C [72]. All subsequent steps were carried out at room temperature (RT: 24–26°C) using (cRPMI). Primary antibody was added at 1∶50 or 1∶100 dilution, and cells were incubated for 1–3 hrs. RBCs were pelleted at 500 *g*, washed and treated with appropriate Alexa 488 or 594 conjugated secondary antibody at 1∶500 to 1∶1000 dilutions (Molecular Probes, invitrogen, USA) for 2 hrs. After washing 3–4 times, cells were incubated for 5 min with DAPI (1 µg/ml) (Roche, Germany, Cat No. 10236276001). Tubulin tracker (Invitrogen, Cat No. T34075) was also used to stain tubulin of the parasite, but this step was carried out in the absence of glutaraldehyde. For permeabilized IFA, both on slides and in solution, fixation method was followed as described above. Post-glutaraldehyde treatment, cells were permeabilized with 0.05 to 0.1% TritonX-100 in PBS for 20 min at RT, and the media used subsequently was PBS containing 0.05 to 0.1% TritonX-100. Cells were treated with secondary antibody conjugated with either Alexa 488 or 594 (Molecular Probe, Invitrogen). To stain the nucleus of the parasite, DAPI (1 µg/ml) was used. Cells were imaged using confocal microscope Exciter LSM500 from Zeiss (Germany). Acquired IFA images were processed by ImageJ software. The primary antibodies used were anti-PfP2 mAbs E2G12 and A12D9 (1∶50 or 1∶100), α-PfP1 polyclonal (1∶1000), anti-PfP0 mAb E5F4 (1∶50); anti-MSP1-19 (1∶1000) (from MR4, USA, MRA-318); anti-β tubulin antibody (1∶1000) (Sigma Aldrich, cat no. T8328); anti-PCNA (1∶2000) (a kind gift from Dr. Suman Dhar, JNU, Delhi, India), Tubulin-tracker (Invitrogen, Cat No. T34075), α-actin antibody (1∶1000) (Phalloidin-Alexa488) (Invitrogen, USA, Cat No. A12379).

### Preparation of *P. falciparum*, *P. berghei* and *P. yoelii* infected RBC ghost and cytosol

Infected RBC at about 10% parasitemia was pelleted at 500 *g* for 5 min, and washed with cRPMI once. RBC pellet was resuspended in 0.15% Saponin and protease inhibitor cocktail (Sigma-Aldrich, Inc, St. Louis, MO, USA, cat. P8340) and 1 mM PMSF in PBS, pH 7.4 for15 min at 37°C. Sample was then centrifuged for 10 min at 10,000 *g* at 4°C to get the parasite pellet, which was sonicated and stored at −80°C. About 60–70% of the opaque supernatant (ghost and cytosol fraction) was gently separated to avoid contamination with the parasite pellet. This supernatant fraction was pelleted at 20,000 *g* for 2 hrs at 4°C, washed twice with PBS, pH 7.4, and stored at −80°C for use as IE ghost. After ghost precipitation, the supernatant (IE cytosol) was stored at −80°C for subsequent analysis. All buffers contained protease inhibitor cocktail (Sigma-Aldrich, Inc, St. Louis, MO, USA, cat. P8340).

### Immunoprecipitation assay (IP)

*P. falciparum (Pf)* and *P. yoelii (Py)* parasite pellets were suspended in 200 µl non-denaturing lysis buffer (20 mM Tris HCl pH 8.0, 137 mM NaCl, 10% glycerol, 1% NP-40, 2 mM EDTA) in the presence of protease inhibitor cocktail (Sigma-Aldrich, Inc, St. Louis, MO, USA, Cat No. P8340) on ice for 15 min. Cells were then briefly sonicated (Branson Sonifier 450, Danbury, C.T) for 1 min, and centrifuged at 15,000 *g* at 4°C. The supernatant was collected, protein was estimated using Bradford reagent (Sigma-Aldrich, Cat No. B6916), and 100 µg protein was incubated with 10 µl packed protein G-sepharose beads (Amersham Biosciences, Sweden, Cat No. 17-6002-35) at 4°C for 1 hr for pre-clearing. Packed Protein G sepharose beads (20 µl) was washed repeatedly and the protein content of the pre-cleared lysate was estimated. For 0.1 mg protein lysate, 0.001 mg of anti-P2 mAb (E2G12) or control pre-immune serum was added to get a final concentration of antibody of 1∶100. The protein-antibody solutions were incubated at 4°C for 6 hrs on a rotary shaker, followed by incubation with 20 µl of packed Protein G-sepharose beads at 4°C for 2 hrs. Subsequently, the beads were centrifuged at 500 *g* and washed 6 times with lysis buffer. To the beads, SDS-PAGE loading buffer was added and boiled for 5 min followed by centrifugation at 15,000 *g* for 15 min at room temperature. The supernatant was loaded on SDS-PAGE for immunoblot. Appropriate gel-bands were cut out and processed for MS analysis.

### Mass spectrometry of proteins by ESI MS/MS (Electronspray Ionization Mass spectrometry/Mass spectrometry)

SDS-PAGE of appropriate protein preparation was carried out and the gel was stained with Coomassie Brilliant Blue R250 (SRL, India) or through silver staining. Gel pieces were cut at the region corresponding to 65 kDa and used for in gel digestion as described below. Gel pieces were washed with 100 mM ammonium bicarbonate for 15 min at 37°C followed by repeated washings with 50% Acetonitrile (ACN) in 100 mM ammonium bicarbonate (buffer A) for 15 min at 37°C, until the gel pieces were completely destained. 10 mM DTT (Sigma-Aldrich, Inc, St. Louis, MO, USA, Cat. No. D0632) in 100 mM ammonium bicarbonate was added and incubated at 56°C for 30 min. Gel pieces were then incubated in 55 mM Iodoacetamide (Sigma-Aldrich, Inc, St. Louis, MO, USA, Cat. No. A3221) prepared in 100 mM ammonium bicarbonate at room temperature for 35 min in the dark, followed by washing once with100 mM ammonium bicarbonate and twice with buffer A. Digestion was set up by the addition of trypsin (Sigma-Aldrich, Inc, St. Louis, MO, USA, Cat. No. T8003) (0.2 ug/ul in 100 mM ammonium bicarbonate) to give a trypsin: protein ratio of 1∶20; incubated at 37°C for 12 hrs. The supernatant was collected without the gel pieces and peptides were extracted in three stages by washing once with 50 mM ammonium bicarbonate and twice with 10% formic acid in 50% ACN. All the extracts were pooled and dried under vacuum. To desalt the peptide preparation, C18 spin columns (Pierce, Rockford, USA) were used as per manufacturer protocol. Briefly, C18 column material or resin was activated using 200 µl of buffer A, and centrifuged to separate resin from ACN. Resins were equilibrated with 200 µl of 5% ACN, 0.5% formic acid (buffer B) and centrifuged again to separate from the ACN/formic acid. This was repeated 3 times. Vacuum dried samples were dissolved in 200 µl of sample buffer containing 2% formic acid in 20% ACN. Samples were loaded on to the resins and subsequently centrifuged to get the supernatant. Again the supernatant was loaded on to the column and repeated 4 times to ensure maximum binding of the peptide to the column material. Resins were washed twice with 200 µl of buffer B. Peptides were eluted using 50 µl 70% ACN. The samples were dried completely under vacuum and suspended in 10 µl of 5% ACN in 0.05% formic acid, prior to MS/MS analysis using ESI MS/MS, nano LC, model no. 6510, Agilent Technologies and data was analyzed using Mascot protein database.

### Treatment of *P. falciparum* cultures with various antibodies

*P. falciparum* culture was synchronized using sorbitol [25], and then subjected to treatments with various anti-P2-mAbs such as E2G12, A12D9; anti-P0 mAb E5F4 and anti-β actin (Sigma). Cultures were also treated with PBS (no antibody treatment), and Sp2/O hybridoma culture medium and mouse pre-immune serum for control treatments. Antibody was precipitated using ammonium sulphate from all mAbs, Sp2/O medium and pre-immune serum, and used at 1 mg/ml concentrations. All the treatment solutions were filter sterilized using 0.22 µm syringe filter (Millipore, Bedford, USA). Haematocrit of *P. falciparum* culture used was typically 2–5%. At appropriate time points, samples from triplicate wells were collected, washed with cRPMI twice and smears were made on glass slides, and remaining cells were pelleted for SIFA, IFA or parasite/IE protein preparation. Counting was performed by staining the nucleus with DAPI and determining the numbers of single nucleated (SN); di-nucleated (DN); tri-nucleated (TN) and MN (multinucleated; >3 nuclei) cells. For statistical analysis, each microscopic field was counted and 100 such fields were used for analysis of the percentage of various cell types. Each field consisted of about 100 cells, of which 8–10 cells were infected.

### Probing RBC surface exposed P2 by flow cytometry of infected erythrocytes

*P. falciparum* 3D7 infected erythrocytes were synchronized and IE from different time points (PMI) were solution stained using anti PfP2 mAb E2G12 (neat culture supernatant) and DAPI (1 µg/ml). Up to 10 million cells were counted using LSR Fortessa (BD Biosciences, USA) and analyzed by FACS DIVA software. Gating strategy has been shown in Figure S4.

### Visualization of tubo-vesicular network (TVN) by BODIPY- TR ceramide staining

BODIPY-TR ceramide (Invitrogen, USA, Cat No. D7540) was used to stain tubo-vesicular network (TVN) as described in [43]–[45]. Nuclei were stained by DAPI (1 µg/ml). Cells were imaged using confocal microscope Exciter LSM500 from Zeiss (Germany). Acquired IFA images were processed by ImageJ software. To assess quantitatively for TVN, the total BODIPY ceramide intensity was determined for each infected cell, from which the fluroscence intensity of the PVM and its internal structures was subtracted. This would allow the determination of fluroscence intensity present only in the RBC cytosol. This value was then further normalized to the intensity of the IE RBC membrane.

### Monitoring lipid import with the lipid marker FM4-64

FM4-64 (N-(3-triethylammoniumpropyl)-4-(6-(4-(diethylamino)- phenyl) hexatrienyl)pyridinium dibromide) (Invitrogen, USA, Cat No. T13320) is a fluorescent lipid marker which was used to monitor endocytic lipid import as described in [42]. Nuclei were stained by DAPI (1 µg/ml). Cells were imaged using confocal microscope Exciter LSM500 from Zeiss (Germany). Acquired IFA images were processed by ImageJ software.

### Monitoring uptake of FM4-64 by Flow cytometry

*P. falciparum* 3D7 infected RBCs of different erythrocytic stages, starting from 6 hrs to 48 hrs PMI, were incubated with 16 µM of FM4-64 for 15 min at 37°C in cRPMI. Cells were washed twice using cRPMI and stained for the nucleus using DAPI. The uptake of FM4-64 was monitored under live cell conditions. The uptake of FM4-64 was measured under different conditions, such as control (no antibody), in the presence of E2G12 (neat culture supernatant) and after removal (washed) of E2G12. About 3–4 million cells were counted in triplicate and monitored for mean fluorescence intensity of FM4-64.

### Statistical analysis

Statistical significance was calculated by two ways ANNOVA with post-test, for all pair of columns. Any pair was considered to be significantly different if they yielded a *p* value<0.05, denoted by *. A *p* value<0.01 was denoted by ** and a *p* value<0.001 was denoted by ***.

## Supporting Information

Figure S1 **ClustalW analysis of ‘P’ proteins.** (**A**) Clustal W of P2 protein across several *Plasmodium* species, exhibiting 75% identity. The bar shows the position of putative transmembrane domain as predicted by TMpred and TMHMM2.0 and TopPred transmembrane domain prediction software from expasy.org (**B**) Amino acid sequence **c**omparison of *P. falciparum* P1, P2 and P0 proteins. Conserved amino acids have been shown in yellow, identical residues in green, similar residues in cyan.
(TIF)

Figure S2 **Immunoblot of parasite, IE ghost and IE cytosol proteins using various antibodies.** *P. falciparum* infected erythrocytes from asynchronous cultures were lysed gently with saponin to separate parasite and the IE components. Immunoblots of 40 µg each of parasite protein lysate (Pf), infected erythrocyte ghost (IE ghost), and infected erythrocyte cytosol (IE cytosol) were probed with α-P2 mAb E2G12, α-β actin mAb and α-MSP1 polyclonal antibody.
(TIF)

Figure S3 **Immunoprecipitation (IP) of** ***P. falciparum*** **parasite crude proteins using E2G12.** IP of parasite crude protein extract was carried out using anti-P2 mAbs E2G12. (**A**) Protein complex was separated on a 12% SDS-PAGE and silver-stained. The 65 kDa band (shown by arrow) was subjected to ESI MS/MS analysis. (**B**) Arrow indicates parent ion and peptide sequence was determined using observed and calculated mass. Data was analyzed using Matrix Science-Mascot database. (**C**) Immunoblot of (I) *P. berghei* and (II) *P. yoelii* infected RBC ghost and RBC cytosol probed using anti-PfP2 mAb E2G12 and anti-spectrin antibody.
(TIF)

Figure S4 **Gating strategy of Flow cytometry.** (**A**) shows the FSC vs SSC plot of all the particles drawn in the flow cytometer (BD Fortessa). Out of these we gated out the single cell population as shown. Further analysis was carried out using only this population. (**B**) Staining pattern of uninfected single cells with DAPI. This was done to set a threshold level of DAPI fluroscence beyond which the cells were considered to be DAPI positive (infected). (**C**) DAPI **s**taining of RBCs with asynchronous stages containing 2% parasitemia. Note that the infected cells show a DAPI fluroscence beyond the threshold set previously. (**D**) Solution staining pattern of uninfected red cells with anti-P2 mAb E2G12. This was done to set a threshold level of fluroscence beyond which the cells were considered to be P2 positive. (**E**) Solution staining of RBCs with asynchronous stages having 2% parasitemia using E2G12. (**F**) Staining of uninfected red cells with FM4-64 to set a threshold level for FM4-64 fluroscence. (**G**) FM4-64 **s**taining of RBCs with 2% parasitemia. The uptake of FM4-64 by infected RBCs was strong with a large shift in MFI.
(TIF)

Figure S5 **Vector map for P2/pSSPF2.** The gene expression of P2-GFP is carried out by two units in the malarial parasite. The first unit is for expressing the recombined gene of interest, P2 (*P2*; turquoise) between *Bgl*II and *Avr*II sites. The GFP tag (*GFP*; green) is downstream of the gene of interest between *Avr*II and *Xho*I (or *Pst*I) sites. The expression of this recombined gene is under the regulation of *P.falciparum* heat shock protein 86 promoter region (*hsp86-5*; pink) and the 3′ sequence of *P. berghei* DHFR-TS gene (*PbDT*-3; teal). The other vital unit for selection of transfectants is the human DHFR gene (*hdhfr*: teal) which confers resistance under drug pressure with the anti-folate WR99210. *P. falciparum* calmodulin promoter (*CAM*-5 ; grey), and the 3′ sequence of the *P. falciparum* histidine-rich protein 2 gene (*hrp2*-3 ; orange) drive the expression of this unit. The two expression units are arranged in head-to-head orientation on either side of the 0.8 kb DNA sequence containing the Rep20 repeats (*Rep20*, yellow). The arrows indicate the direction of transcription in each expression unit. Unique restriction sites *AvrII*, *Bam*HI, *Bgl*II, *Eco*RI, *Hin*dIII, *Not*I, *Pst*I and *Xho*I are indicated. The plasmid backbone was derived from the *E. coli* vector pGEM [71].
(TIF)

Figure S6 **Arrest of** ***P. falciparum*** **infected erythrocytes using anti-P2 mAb (A12D9).** (**A**)**.** Synchronized *P. falciparum* cells were treated with A12D9 mAb for 24 hrs starting from 12 to 36 hrs PMI. At 36 hrs the arrested cells were washed and split into two flasks and cultured for further 24 hrs with and without A12D9 (antibody continued and removed, respectively). The % IE was scored using DAPI at 36 hrs, and after another 24 hrs post washing; corresponding to 60 hrs PMI. (**B and C**)**.** Representative images for the DAPI stained cells showing control and arrested cells in the presence of A12D9 antibodies. Scale bar indicates 2 µm.
(TIF)

Figure S7 ***P. falciparum*** **growth inhibition in culture using anti-P2 mAb (E2G12).** (**A**)**.** Synchronized *P. falciaprum* infected RBCs at 8% parasitemia were treated with anti-P2 mAb (E2G12) or Sp2/O at 1 mg/ml from 12 to 60 hrs. Sp2/O is the hybridoma cell culture supernatant which was ammonium sulfate precipitated the same way as the E2G12 mAb supernatant. Parasitemia was measured through Geimsa staining at 48 hrs and at 60 hrs. Results are represented as a percentage change in comparison with the starting 8% parasitemia. For each time point, about 7000 cells were counted. **p<0.01. n = 5 (**B**) Percent parasitemia change in comparison with the starting parasitemia at different time points in parasite development for control (no antibody) and E2G12 treated cells as determined through flow cytometric analysis using DAPI stain. *p<0.05.
(TIF)

Figure S8 **Flow Cytometry dot-plots of surface PfP2 and DAPI Staining.** Representative flow cytometric data of *P. falciparum* infected synchronously cultured cells, double stained with E2G12 and DAPI, at various stages of development. The stretch of DAPI positive cell population is in quadrant 4 and P2/DAPI double positive cells are in quadrant 2. The percentages mentioned in Q2 and Q4 are for DAPI positive infected cells only. Panels **A–D** show dot-plots for control infected RBCs without antibody at **A**: 12 hrs; **B**: 30 hrs; **C**: 36 hrs; and **D**: 48 hrs in the erythrocytic cycle, while Panels **E–G** show dot-plots of infected RBCs incubated with anti P2 mAb (E2G12) at **E**: 30 hrs; **F**: 36 hrs and **G**:48 hrs PMI. The mAb was added at 12 hrs PMI. The total number of DAPI positive cells decrease considerably by 48 hrs in the presence of E2G12.
(TIF)

Figure S9 **Flow Cytometry histograms of PfP2 Staining.** Representative flow cytometric frequency histograms of PfP2 stained *P. falciparum* infected RBCs at various time points PMI. During the acquisition of such data, only the infected cells were gated out through DAPI staining, and appropriate cutoff was marked for P2 positivity (as shown in fig. S4). **A:** P2 stained control infected RBCs without any antibody treatment; **B** with anti-P2 mAb (E2G12) added at 12 hrs; **C** with anti-P2 mAb (E2G12) added at 12 hrs and washed off at 36 hrs, monitored at 42 and 48 hrs PMI.
(TIF)

Figure S10 **Growth inhibition and synchronization of** ***P. yoelii*** **infected erythrocytes using anti-PfP2 mAb E2G12 in culture.** *P. yoelii* infected erythrocytes (IE) were incubated with E2G12 for 24 hrs; washed thoroughly with cRPMI, split into two sets and cultured further for 11 hrs in cRPMI, with (Ab present) or without (Ab removed) mAb E2G12. (**A**) Cells were removed at different time points and solution immunofluorescence (SIFA) was performed. (**B**) DAPI images of IE after 11 hrs of culturing; E2G12 removed (control) and E2G12 continued. (**C**) Shows the representation of single nucleated (SN), di-nuclear (DN); tri-nuclear (TN) and >3 nuclei (MN) cells in the population, as determined through DAPI staining of infected RBC population up to11 hrs in the absence (C-Upper: Ab removed) and presence of E2G12 (C-Lower: Ab present). (**D**) Upper panel shows SIFA of live *P. yoelii* infected RBC using E2G12 (red) and Tubulin Tracker (green) and Lower panel shows IFA of *P. yoelii* infected RBCs cultured with E2G12 for 32 hrs using E2G12 (green) and antibodies against proliferating cell nuclear antigen (PCNA) and tubulin (red). *P<0.05, **P<0.01. Significance of each data point was calculated with respect to the previous data point. n = 5. Scale bar indicates 2 µm.
(TIF)

Figure S11 **BODIPY-ceramide staining of Tubovesicular network (TVN) of** ***P. falciparum*** **and** ***P. berghei*** **infected RBCs in the presence of mouse pre-immune sera, anti-P2 mAbs (A12D9 and E2G12) and after removal of E2G12.** Representative images of BODIPY-ceramide staining of *P. falciparum* and *P. berghei* infected RBCs after various treatments; *P. falciparum* infected (**A**)**:** control cells; (**B**)**:** cells treated with A12D9. Antibodies were added at 12 hrs and the cells were observed at 24 and 36 hrs. (**C**)**:** *P. berghei* and (**D**)**:** *P. falciparum* infected cells treated with control (**CI**) mouse pre-immune sera; (**DI**) no antibody; (**CII, DII**) with E2G12 continued and (**CIII, DIII**) after removal of E2G12, monitored after 4 hrs post-washing. Scale bar indicates 2 µm.
(TIF)

Table S1 **Mass spectrometric data of immunoprecipitated 65 kDa band excised from SDS-PAGE of IE ghost preparation from IEs at 30 hrs PMI.** List of peptides of P2 and other proteins was obtained through mascot analysis and is shown with all mass spectrometric parameters.
(XLS)

Table S2 **Mass spectrometric data of immuno-precipitated** ***P. falciparum*** **parasite protein using E2G12 (Figure. S3A).** List of peptides of P2 and other proteins was obtained through mascot analysis and is shown with all mass spectrometric parameters.
(XLS)

Video S1 **3D reconstruction of confocal images of tubovesicular network stained using BODIPY- ceramide in** ***P. falciparum*** **infected RBC showing the mesh like structures.**
(AVI)

Video S2 **3D reconstruction of confocal images of tubovesicular network stained using BODIPY-ceramide in** ***P. falciparum*** **infected RBC treated with anti-PfP2 mAbs E2G12, showing the absence of mesh like structures.**
(AVI)
