# Correction: Arrest of Nuclear Division in *Plasmodium* through Blockage of Erythrocyte Surface Exposed Ribosomal Protein P2

**DOI:** 10.1371/annotation/913cb443-4033-4841-8666-1d348949a010

**Published:** 2012-08-21

**Authors:** Sudipta Das, Himanish Basu, Reshma Korde, Rita Tewari, Shobhona Sharma

There are three incorrect references in this manuscript:

The last sentence for panel C of the Figure 1 legend should have cited reference 39 instead of 36. 

The correct sentence should read: (a) Coomassie stain of the gel; (b), (c) and (d) are immunoblots probed with (b) α-P0 mAb E5F4 [39]; (c) α-P2 mAb A12D9; and (d) α-P2 mAb E2G12.

In the legend of figure 6, the structure of TVN should be cited 43 and 44 instead of 41 and 42.

The correct sentence should read: Cells were harvested from 24 hrs to 36 hrs at various time points, washed, incubated with BODIPY-ceramide for 15 min at 37°C, and the structure of TVN [43], [44] was recorded in IEs.

