# Supplementary figures and images for: Arrest of Nuclear Division in Plasmodium through Blockage of Erythrocyte Surface Exposed Ribosomal Protein P2
Source: PLoS Pathog. 2012 Aug 9;8(8):e1002858. doi: 10.1371/journal.ppat.1002858 (PMC3415463; doi:10.1371/journal.ppat.1002858)

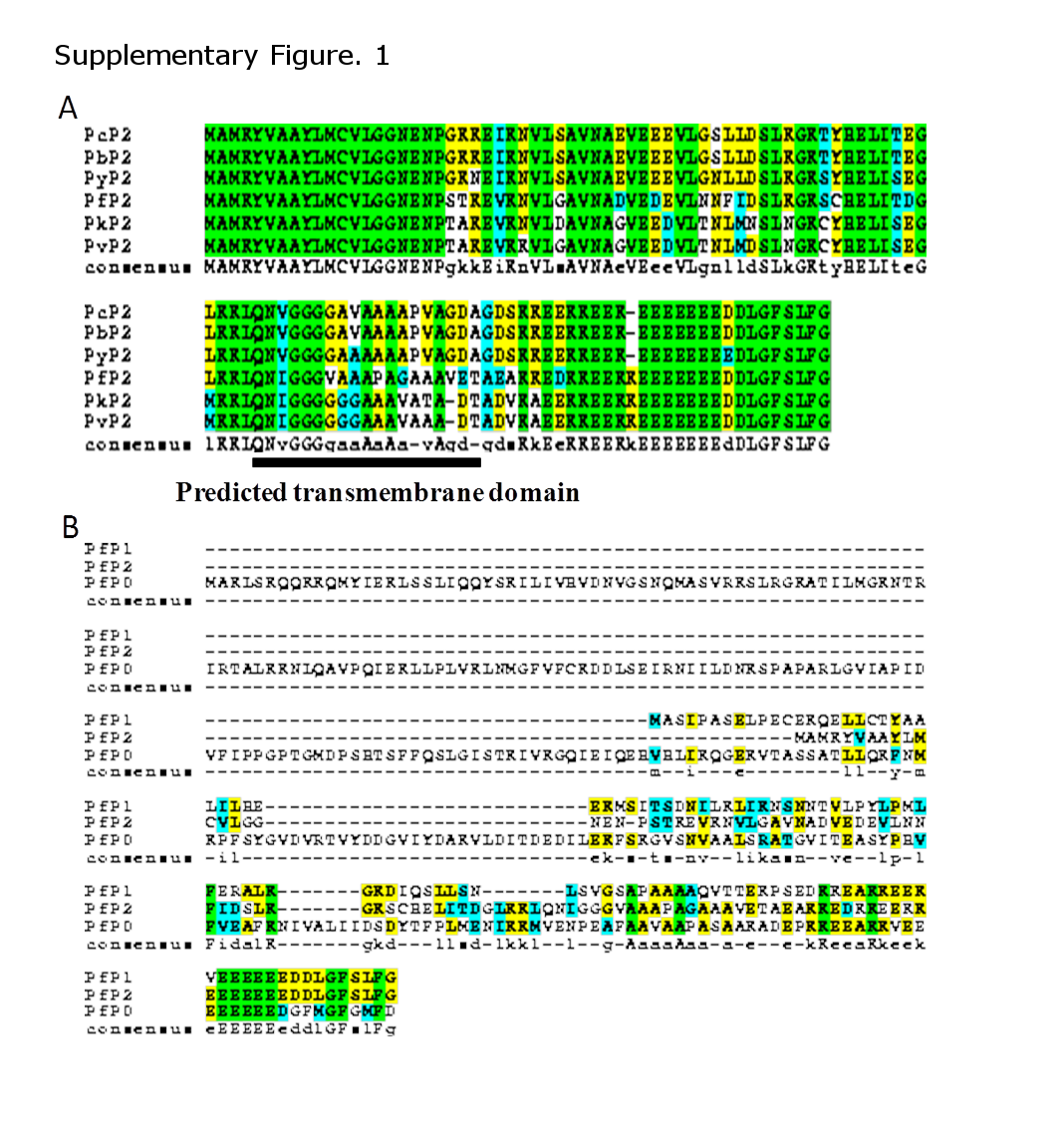

Supplement: Figure S1 — ClustalW analysis of ‘P’ proteins. (A) Clustal W of P2 protein across several Plasmodium species, exhibiting 75% identity. The bar shows the position of putative transmembrane domain as predicted by TMpred and TMHMM2.0 and TopPred transmembrane domain prediction software from expasy.org (B) Amino acid sequence comparison of P. falciparum P1, P2 and P0 proteins. Conserved amino acids have been shown in yellow, identical residues in green, similar residues in cyan. (TIF) [file ppat.1002858.s001.tif]

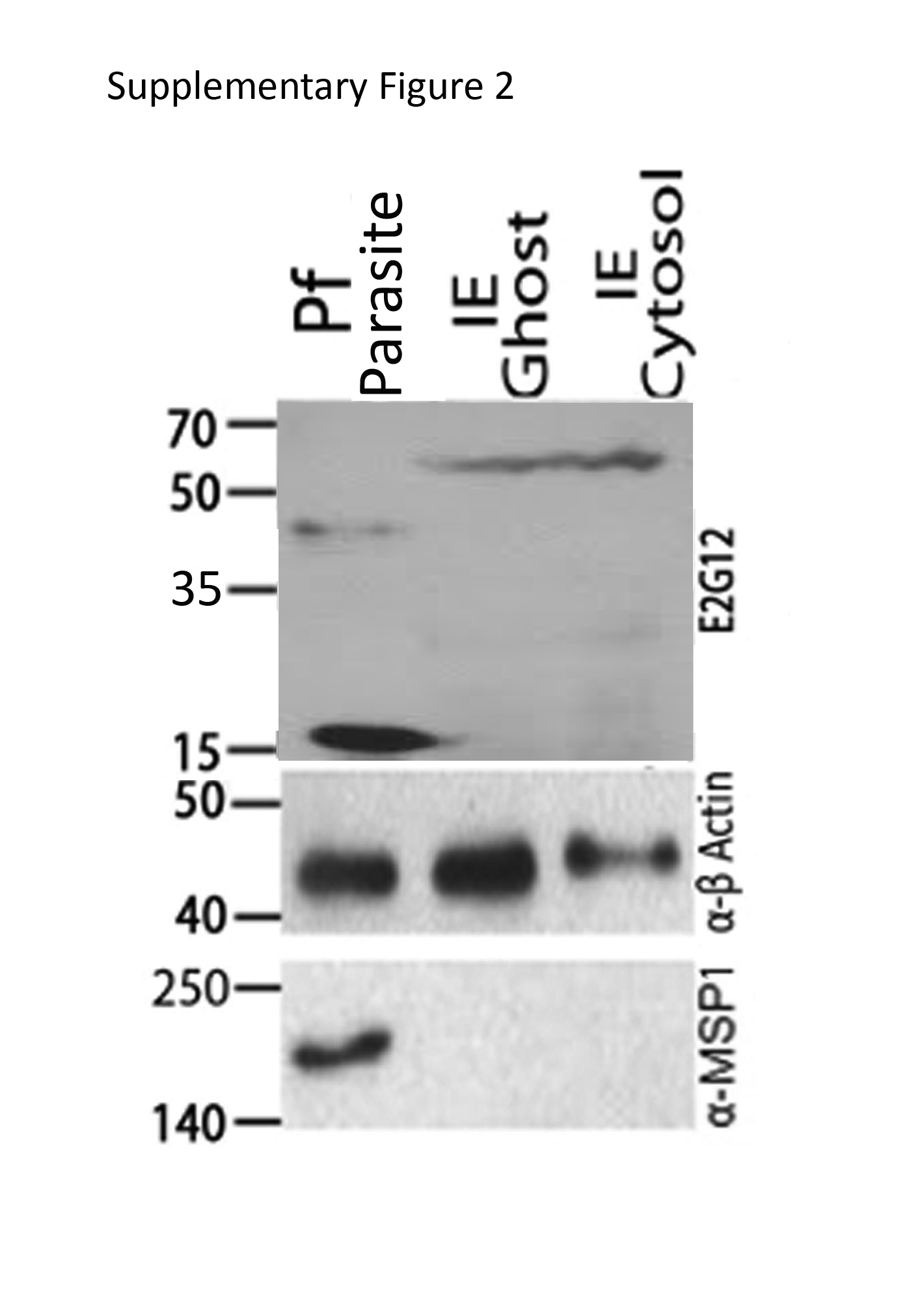

Supplement: Figure S2 — Immunoblot of parasite, IE ghost and IE cytosol proteins using various antibodies. P. falciparum infected erythrocytes from asynchronous cultures were lysed gently with saponin to separate parasite and the IE components. Immunoblots of 40 µg each of parasite protein lysate (Pf), infected erythrocyte ghost (IE ghost), and infected erythrocyte cytosol (IE cytosol) were probed with α-P2 mAb E2G12, α-β actin mAb and α-MSP1 polyclonal antibody. (TIF) [file ppat.1002858.s002.tif]

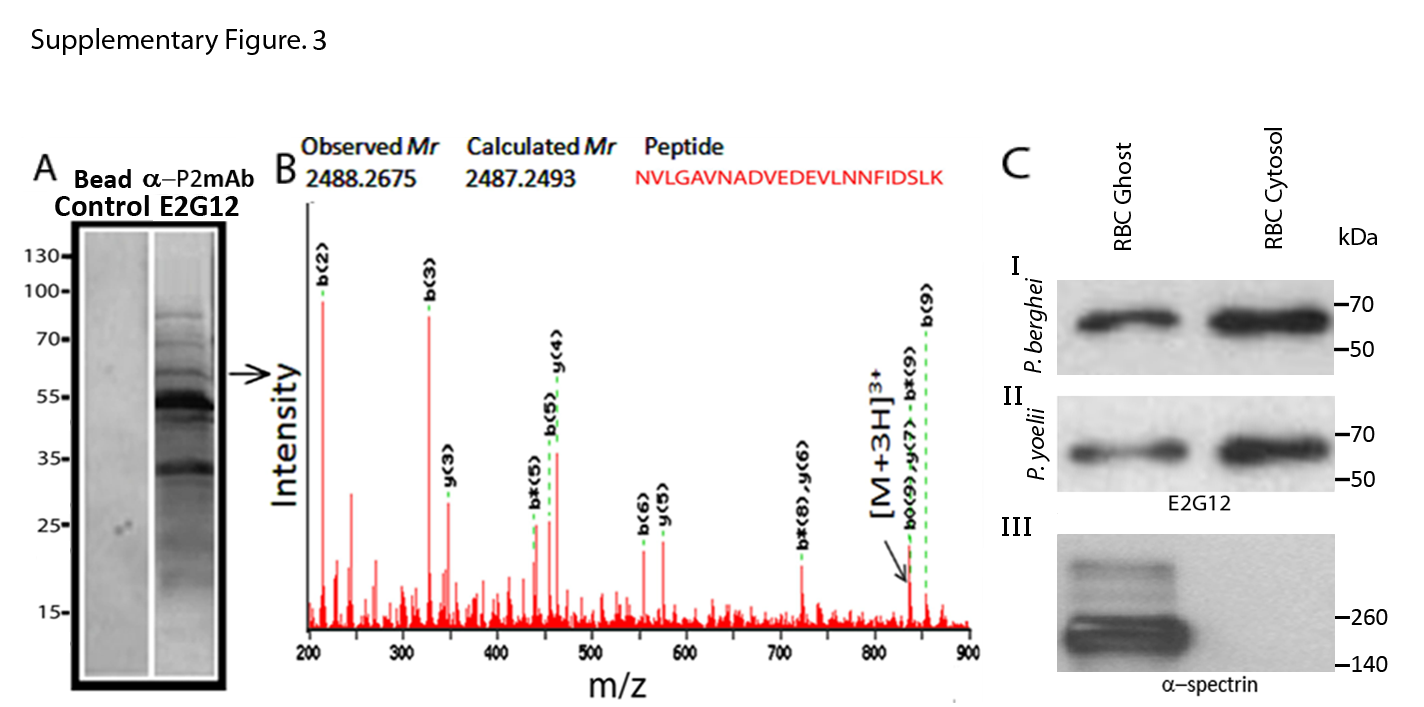

Supplement: Figure S3 — Immunoprecipitation (IP) of P. falciparum parasite crude proteins using E2G12. IP of parasite crude protein extract was carried out using anti-P2 mAbs E2G12. (A) Protein complex was separated on a 12% SDS-PAGE and silver-stained. The 65 kDa band (shown by arrow) was subjected to ESI MS/MS analysis. (B) Arrow indicates parent ion and peptide sequence was determined using observed and calculated mass. Data was analyzed using Matrix Science-Mascot database. (C) Immunoblot of (I) P. berghei and (II) P. yoelii infected RBC ghost and RBC cytosol probed using anti-PfP2 mAb E2G12 and anti-spectrin antibody. (TIF) [file ppat.1002858.s003.tif]

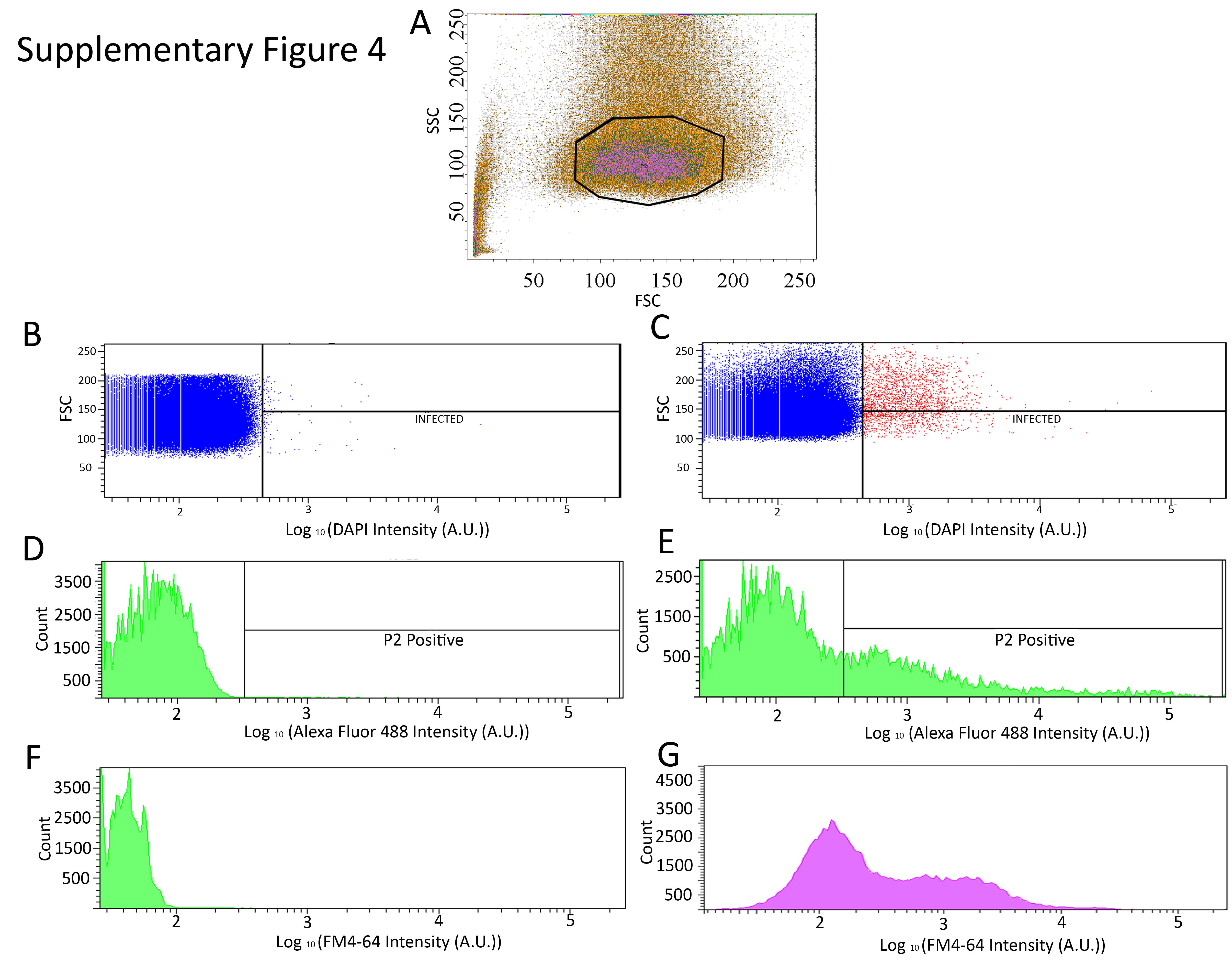

Supplement: Figure S4 — Gating strategy of Flow cytometry. (A) shows the FSC vs SSC plot of all the particles drawn in the flow cytometer (BD Fortessa). Out of these we gated out the single cell population as shown. Further analysis was carried out using only this population. (B) Staining pattern of uninfected single cells with DAPI. This was done to set a threshold level of DAPI fluroscence beyond which the cells were considered to be DAPI positive (infected). (C) DAPI staining of RBCs with asynchronous stages containing 2% parasitemia. Note that the infected cells show a DAPI fluroscence beyond the threshold set previously. (D) Solution staining pattern of uninfected red cells with anti-P2 mAb E2G12. This was done to set a threshold level of fluroscence beyond which the cells were considered to be P2 positive. (E) Solution staining of RBCs with asynchronous stages having 2% parasitemia using E2G12. (F) Staining of uninfected red cells with FM4-64 to set a threshold level for FM4-64 fluroscence. (G) FM4-64 staining of RBCs with 2% parasitemia. The uptake of FM4-64 by infected RBCs was strong with a large shift in MFI. (TIF) [file ppat.1002858.s004.tif]

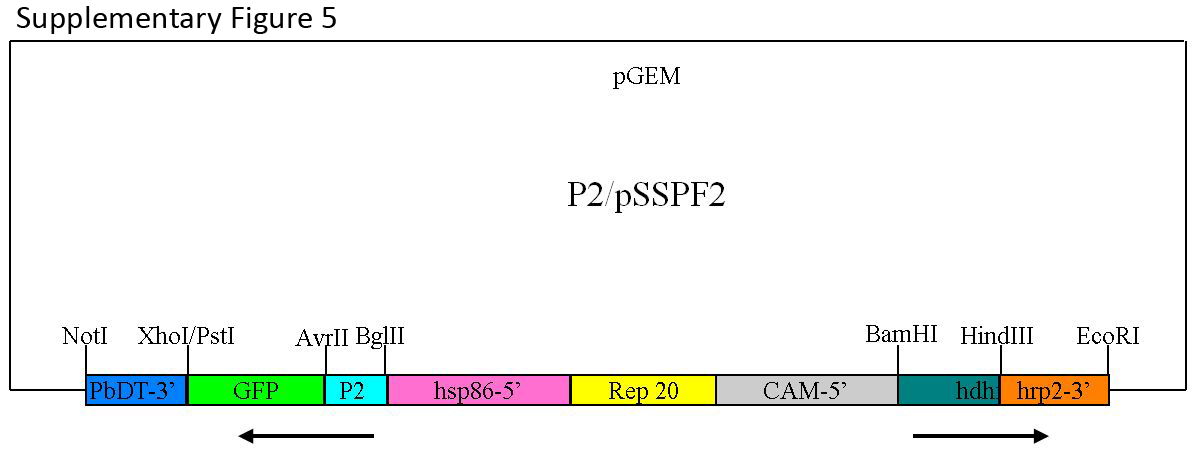

Supplement: Figure S5 — Vector map for P2/pSSPF2. The gene expression of P2-GFP is carried out by two units in the malarial parasite. The first unit is for expressing the recombined gene of interest, P2 (P2; turquoise) between BglII and AvrII sites. The GFP tag (GFP; green) is downstream of the gene of interest between AvrII and XhoI (or PstI) sites. The expression of this recombined gene is under the regulation of P.falciparum heat shock protein 86 promoter region (hsp86-5; pink) and the 3′ sequence of P. berghei DHFR-TS gene (PbDT-3; teal). The other vital unit for selection of transfectants is the human DHFR gene (hdhfr: teal) which confers resistance under drug pressure with the anti-folate WR99210. P. falciparum calmodulin promoter (CAM-5 ; grey), and the 3′ sequence of the P. falciparum histidine-rich protein 2 gene (hrp2-3 ; orange) drive the expression of this unit. The two expression units are arranged in head-to-head orientation on either side of the 0.8 kb DNA sequence containing the Rep20 repeats (Rep20, yellow). The arrows indicate the direction of transcription in each expression unit. Unique restriction sites AvrII, BamHI, BglII, EcoRI, HindIII, NotI, PstI and XhoI are indicated. The plasmid backbone was derived from the E. coli vector pGEM [71]. (TIF) [file ppat.1002858.s005.tif]

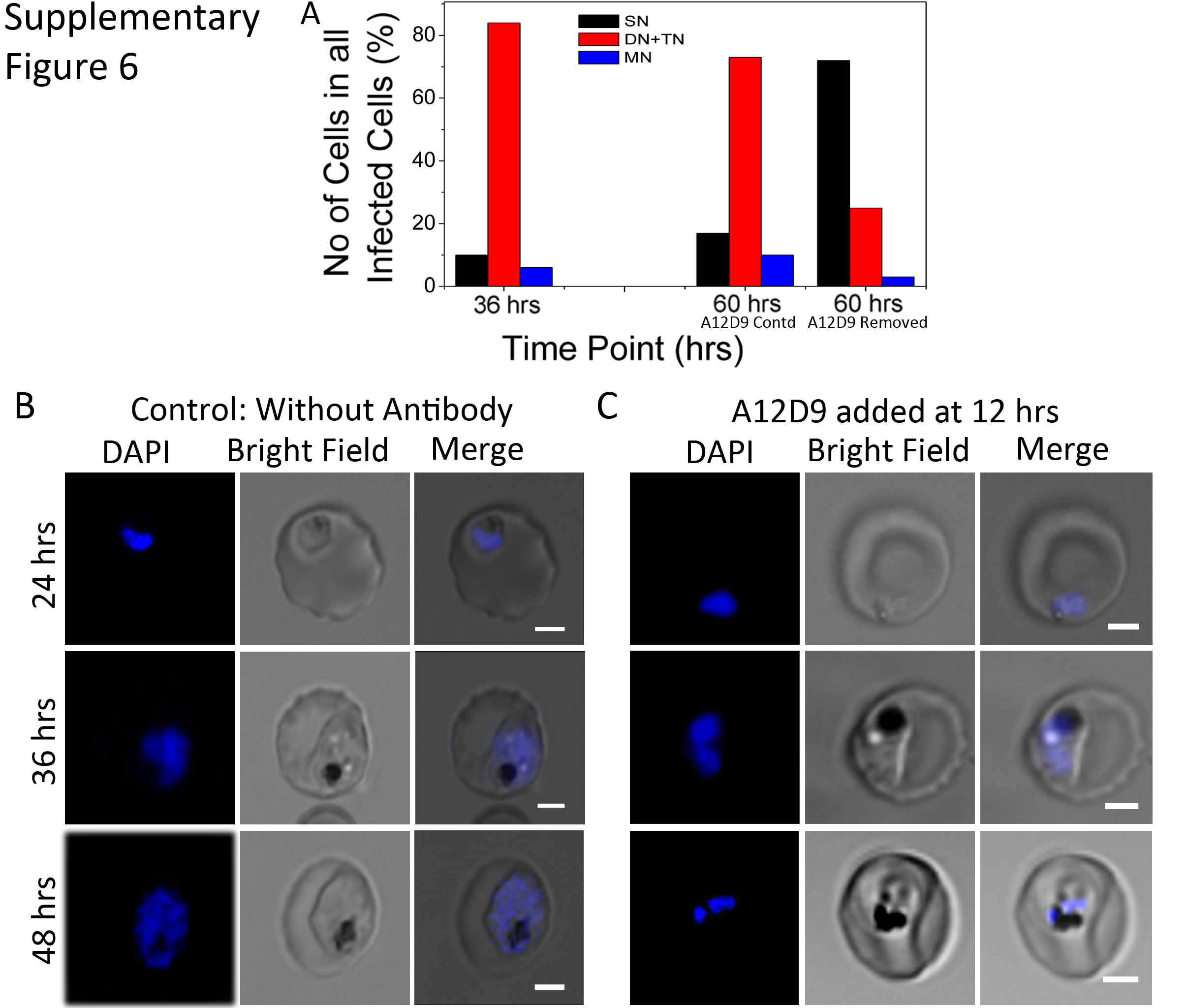

Supplement: Figure S6 — Arrest of P. falciparum infected erythrocytes using anti-P2 mAb (A12D9). (A). Synchronized P. falciparum cells were treated with A12D9 mAb for 24 hrs starting from 12 to 36 hrs PMI. At 36 hrs the arrested cells were washed and split into two flasks and cultured for further 24 hrs with and without A12D9 (antibody continued and removed, respectively). The % IE was scored using DAPI at 36 hrs, and after another 24 hrs post washing; corresponding to 60 hrs PMI. (B and C). Representative images for the DAPI stained cells showing control and arrested cells in the presence of A12D9 antibodies. Scale bar indicates 2 µm. (TIF) [file ppat.1002858.s006.tif]

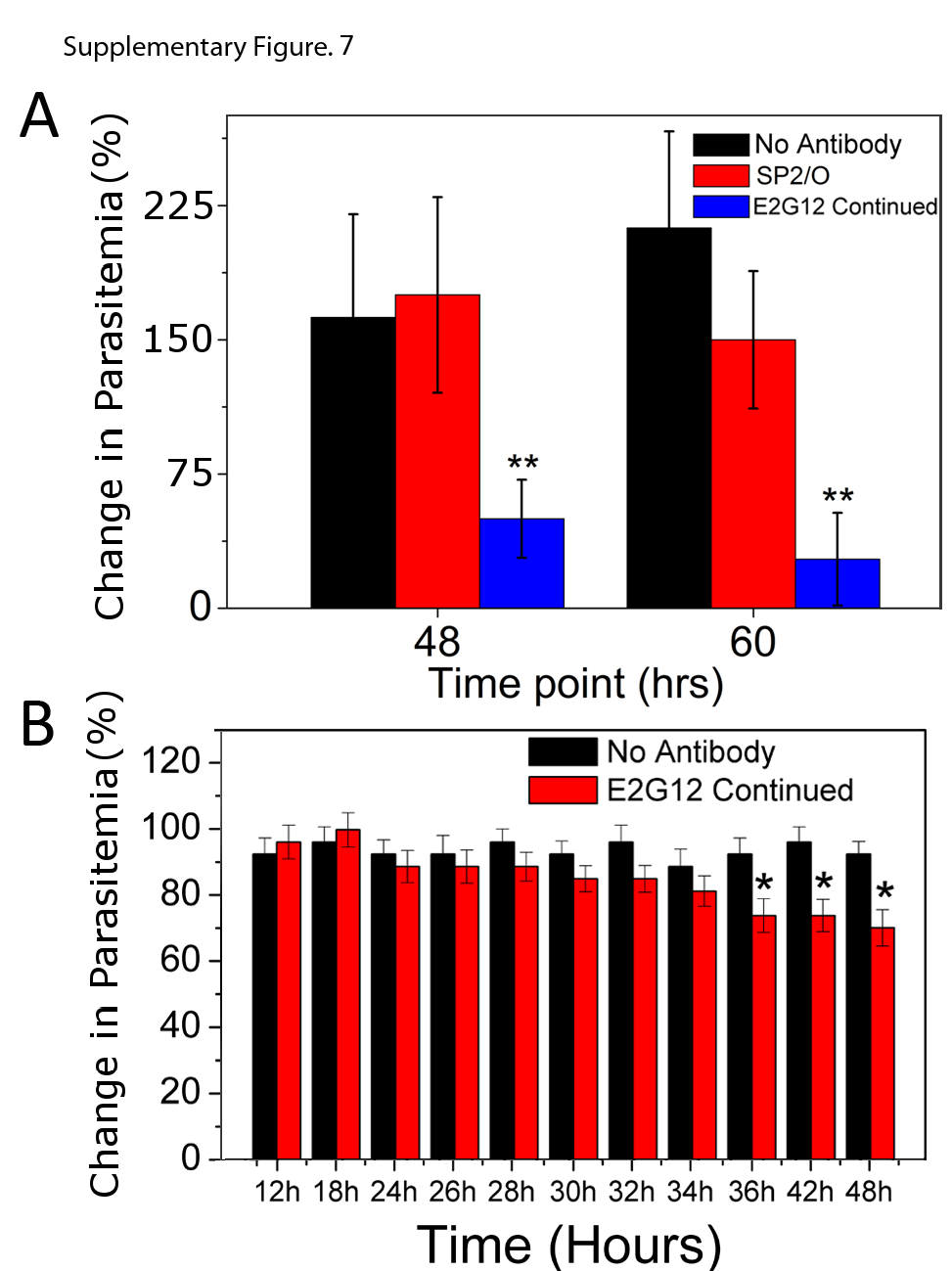

Supplement: Figure S7 — P. falciparum growth inhibition in culture using anti-P2 mAb (E2G12). (A). Synchronized P. falciaprum infected RBCs at 8% parasitemia were treated with anti-P2 mAb (E2G12) or Sp2/O at 1 mg/ml from 12 to 60 hrs. Sp2/O is the hybridoma cell culture supernatant which was ammonium sulfate precipitated the same way as the E2G12 mAb supernatant. Parasitemia was measured through Geimsa staining at 48 hrs and at 60 hrs. Results are represented as a percentage change in comparison with the starting 8% parasitemia. For each time point, about 7000 cells were counted. **p<0.01. n = 5 (B) Percent parasitemia change in comparison with the starting parasitemia at different time points in parasite development for control (no antibody) and E2G12 treated cells as determined through flow cytometric analysis using DAPI stain. *p<0.05. (TIF) [file ppat.1002858.s007.tif]

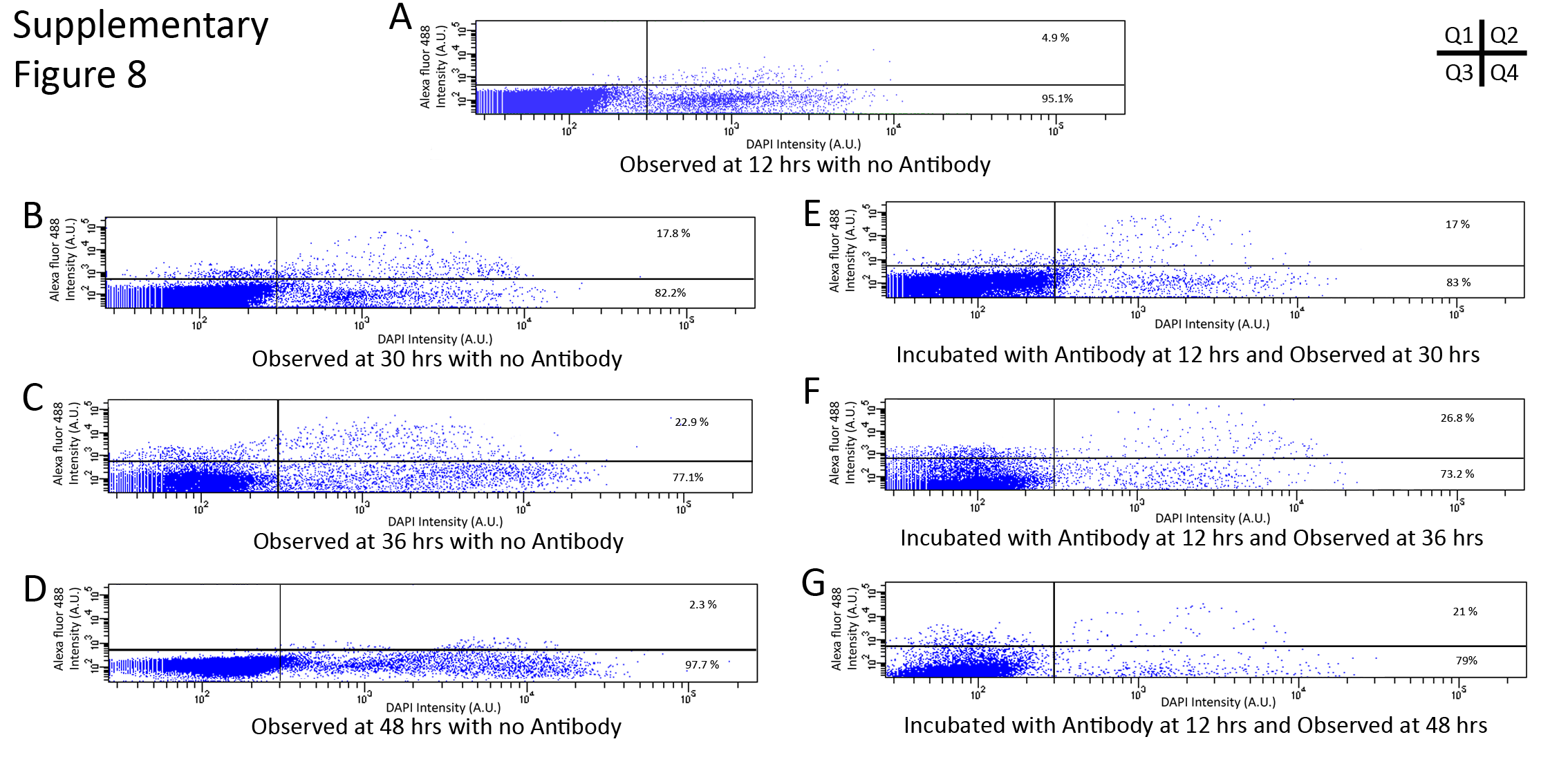

Supplement: Figure S8 — Flow Cytometry dot-plots of surface PfP2 and DAPI Staining. Representative flow cytometric data of P. falciparum infected synchronously cultured cells, double stained with E2G12 and DAPI, at various stages of development. The stretch of DAPI positive cell population is in quadrant 4 and P2/DAPI double positive cells are in quadrant 2. The percentages mentioned in Q2 and Q4 are for DAPI positive infected cells only. Panels A–D show dot-plots for control infected RBCs without antibody at A: 12 hrs; B: 30 hrs; C: 36 hrs; and D: 48 hrs in the erythrocytic cycle, while Panels E–G show dot-plots of infected RBCs incubated with anti P2 mAb (E2G12) at E: 30 hrs; F: 36 hrs and G:48 hrs PMI. The mAb was added at 12 hrs PMI. The total number of DAPI positive cells decrease considerably by 48 hrs in the presence of E2G12. (TIF) [file ppat.1002858.s008.tif]

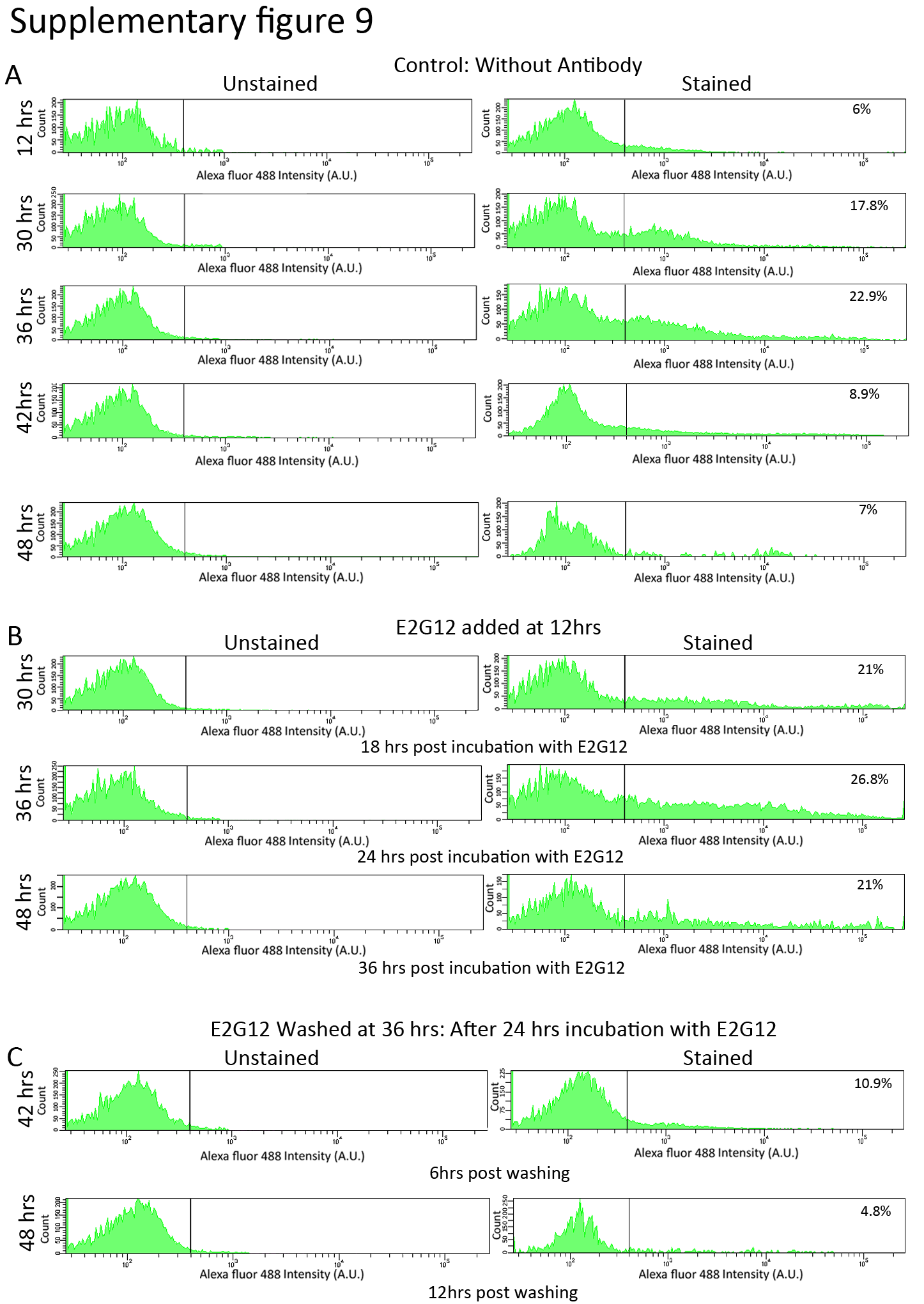

Supplement: Figure S9 — Flow Cytometry histograms of PfP2 Staining. Representative flow cytometric frequency histograms of PfP2 stained P. falciparum infected RBCs at various time points PMI. During the acquisition of such data, only the infected cells were gated out through DAPI staining, and appropriate cutoff was marked for P2 positivity (as shown in fig. S4). A: P2 stained control infected RBCs without any antibody treatment; B with anti-P2 mAb (E2G12) added at 12 hrs; C with anti-P2 mAb (E2G12) added at 12 hrs and washed off at 36 hrs, monitored at 42 and 48 hrs PMI. (TIF) [file ppat.1002858.s009.tif]

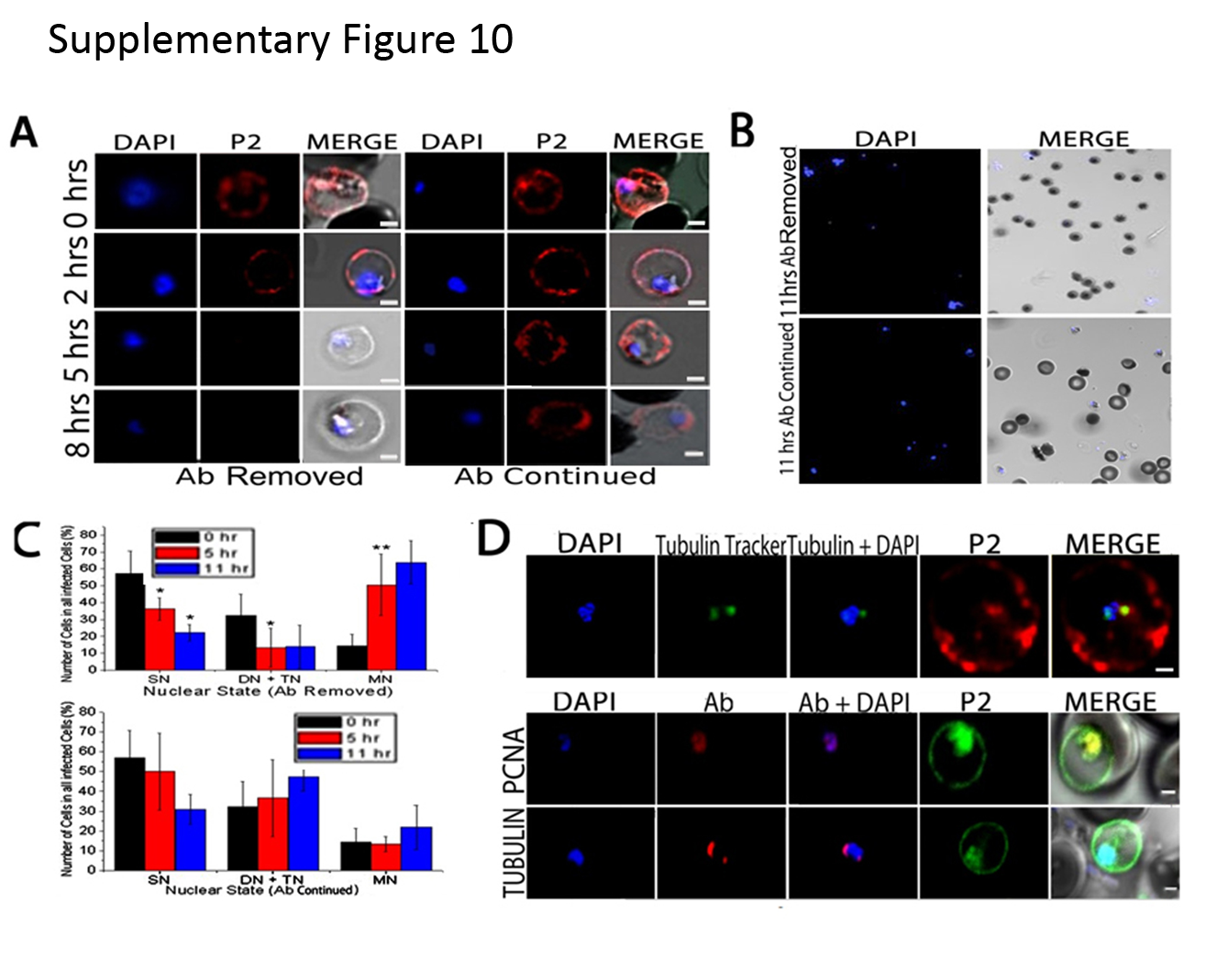

Supplement: Figure S10 — Growth inhibition and synchronization of P. yoelii infected erythrocytes using anti-PfP2 mAb E2G12 in culture. P. yoelii infected erythrocytes (IE) were incubated with E2G12 for 24 hrs; washed thoroughly with cRPMI, split into two sets and cultured further for 11 hrs in cRPMI, with (Ab present) or without (Ab removed) mAb E2G12. (A) Cells were removed at different time points and solution immunofluorescence (SIFA) was performed. (B) DAPI images of IE after 11 hrs of culturing; E2G12 removed (control) and E2G12 continued. (C) Shows the representation of single nucleated (SN), di-nuclear (DN); tri-nuclear (TN) and >3 nuclei (MN) cells in the population, as determined through DAPI staining of infected RBC population up to11 hrs in the absence (C-Upper: Ab removed) and presence of E2G12 (C-Lower: Ab present). (D) Upper panel shows SIFA of live P. yoelii infected RBC using E2G12 (red) and Tubulin Tracker (green) and Lower panel shows IFA of P. yoelii infected RBCs cultured with E2G12 for 32 hrs using E2G12 (green) and antibodies against proliferating cell nuclear antigen (PCNA) and tubulin (red). *P<0.05, **P<0.01. Significance of each data point was calculated with respect to the previous data point. n = 5. Scale bar indicates 2 µm. (TIF) [file ppat.1002858.s010.tif]

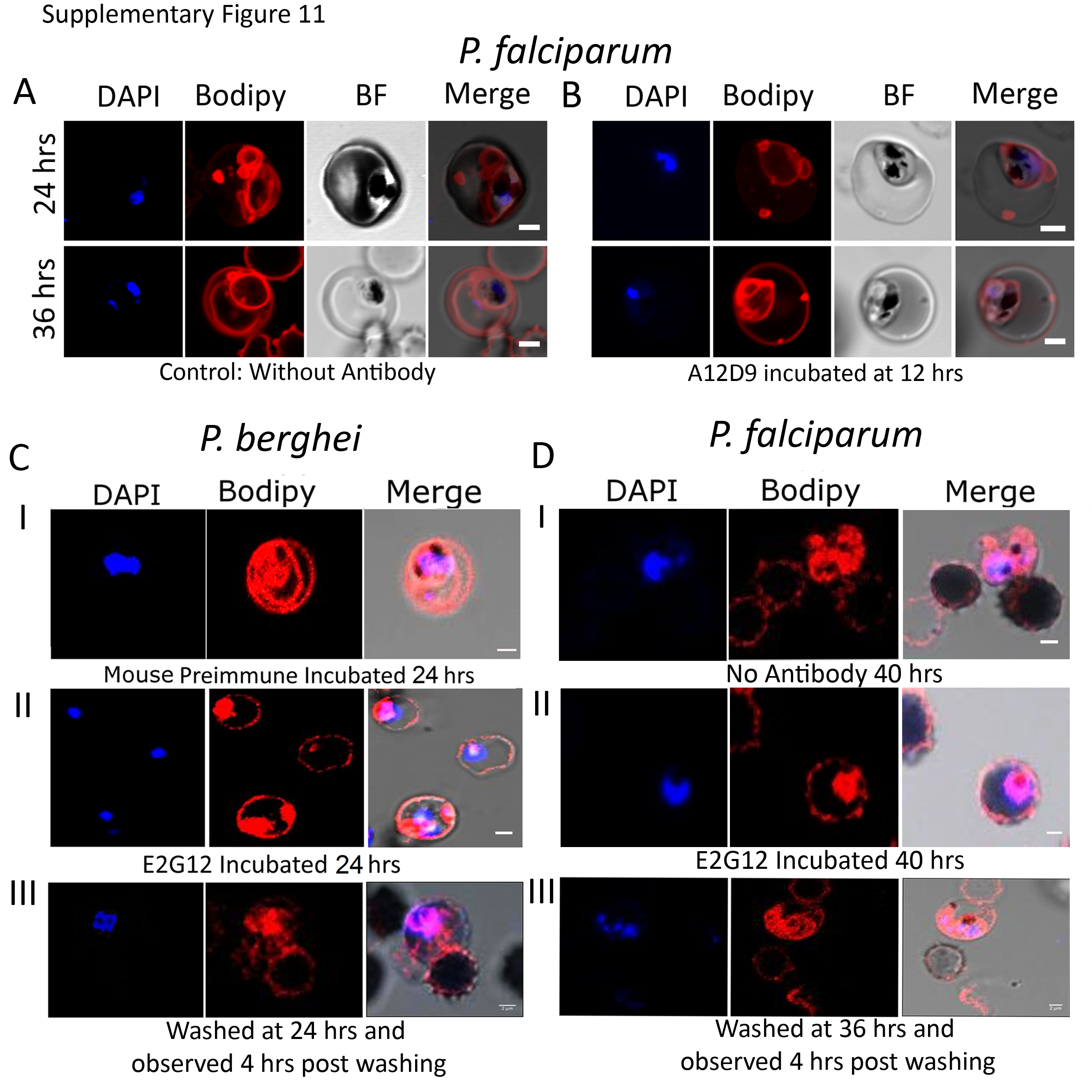

Supplement: Figure S11 — BODIPY-ceramide staining of Tubovesicular network (TVN) of P. falciparum and P. berghei infected RBCs in the presence of mouse pre-immune sera, anti-P2 mAbs (A12D9 and E2G12) and after removal of E2G12. Representative images of BODIPY-ceramide staining of P. falciparum and P. berghei infected RBCs after various treatments; P. falciparum infected (A): control cells; (B): cells treated with A12D9. Antibodies were added at 12 hrs and the cells were observed at 24 and 36 hrs. (C): P. berghei and (D): P. falciparum infected cells treated with control (CI) mouse pre-immune sera; (DI) no antibody; (CII, DII) with E2G12 continued and (CIII, DIII) after removal of E2G12, monitored after 4 hrs post-washing. Scale bar indicates 2 µm. (TIF) [file ppat.1002858.s011.tif]
